# The Members of the Highly Diverse *Crassostrea gigas* Integrin Family Cooperate for the Generation of Various Immune Responses

**DOI:** 10.3389/fimmu.2020.01420

**Published:** 2020-07-23

**Authors:** Zhao Lv, Limei Qiu, Weilin Wang, Zhaoqun Liu, Qing Liu, Lingling Wang, Linsheng Song

**Affiliations:** ^1^Key Laboratory of Experimental Marine Biology, Institute of Oceanology, Chinese Academy of Sciences, Qingdao, China; ^2^University of Chinese Academy of Sciences, Beijing, China; ^3^Center for Ocean Mega-Science, Chinese Academy of Sciences, Qingdao, China; ^4^Laboratory of Marine Fisheries Science and Food Production Processes, Qingdao National Laboratory for Marine Science and Technology, Qingdao, China; ^5^Liaoning Key Laboratory of Marine Animal Immunology, Dalian Ocean University, Dalian, China; ^6^Liaoning Key Laboratory of Marine Animal Immunology and Disease Control, Dalian Ocean University, Dalian, China

**Keywords:** *Crassostrea gigas*, integrin family, immune function, ECM ligand binding, functional division and cooperation

## Abstract

Studies on invertebrate immune receptors can provide insights into characteristics specific to innate immune system. Here, eight α and three β integrins are identified from an invertebrate, the Pacific oyster *Crassostrea gigas*, and their possible immune functions are studied. Oyster α/β integrins exhibit a higher degree of sequence and structural variability than the members from *Homo sapiens* and *Drosophila melanogaster*. The analysis reveals that oyster RGD- and laminin-binding receptor homologs are present in the phylogenetic tree of α integrins, but the other six oyster α integrins mainly form a species-specific branch; meanwhile, oyster β integrins are clustered with insect β integrins but distinct from a member from the mollusk *Biomphalaria glabrata*. Although phylogenetically lacking the important α integrin branches of LDV-binding, PS3-type, and αI-containing integrins, oyster integrins can bind to most ECM ligands, including RGDCP, LDVCP, GFOGERCP, and laminin protein in a distinct binding pattern. Besides, oyster integrins are distributed in different hemocyte subpopulations, while only specific integrins are selectively involved in hemocyte phagocytosis, migration, and encapsulation, and some of them participate in more than one immune response in a sophisticated pattern. Especially, oyster β integrins are arranged in the core to mediate complex immune responses, unlike the counterparts in humans that mainly depend on αI-containing integrins to incite immune reactions. This study represents the first comprehensive attempt to reveal the structural and evolutionary features of the integrin family and their involvement in cellular immune responses in the non-model invertebrate *C. gigas* and sheds light on the characteristics specific to the innate immune system in the integrin family.

## Introduction

Integrins are α-β heterodimeric cell surface receptors that exert various cell adhesion-related functions in multiple biological processes (1–5). They are ancient molecules that may have evolved during the transition of unicellular life forms into multicellular organisms, and therefore are thought to be key factors in the evolution of multicellular organisms (2, 6, 7). Consequently, integrins from different animal phyla have been characterized (8, 9), and almost every species has more than one integrin, all of which together constitute a large gene family with a high variability. In invertebrates, information about the structure, classification, and function of integrin family members is mainly obtained from five phyla, that is, sponges, cnidarians, nematodes, arthropods, and echinoderms (7). Although the fundamental functions and structures of these integrins appear to be conserved, it is likely that every invertebrate species has a specific repertoire of integrins with complex roles and specific cell functions, attributable to the unique and complicated environments in which they survive (10–13). For instance, all αPS3 integrins have been observed to occur only in arthropods, probably because of the arthropod-specific radiation (14). Similarly, the phylogenetic clustering of invertebrate β integrins results in the development of obvious phylum-lineage features, such as the insecta βV and cnidarian β integrin branches, which can be found only in certain animals (14, 15). In specific invertebrate species, these distinct branches of integrins harbor special structures and possibly enable the development of unique functional characteristics that are important for animal survival (7). Although similar examples can be observed in other non-model invertebrates, the relevant data are scattered and not well-summarized. Hence, the evolutionary history of integrins is still obscure, and information about this versatile receptor family in invertebrates still needs to be thoroughly expatiated.

Integrin receptors mediate adhesive events that are vital to generate specific and effective immune responses (3, 16). For example, the integrin-dependent interactions of lymphocytes and antigen-presenting cells with endothelia mainly rely on specific αI-domain containing integrins that are leukocyte-specific receptors (16). The migration of leukocytes to infection sites is critical for immune surveillance and host defense, which greatly depends on the cell adhesion process and is mediated by αLβ2, αDβ2, αMβ2, and αXβ2 (17). In addition, αMβ2 and αXβ2, known as complement receptor type 3 (CR3) and CR4, respectively, also serve as phagocytic receptors in macrophages for the complement-opsonization of foreign particles, which suggests their dual roles in immunocyte migration and phagocytosis in humans (18, 19). Emerging evidences have revealed that invertebrate integrin family members are smaller than those found in humans but have highly varied sequences, special structures, and α-β pairings (7, 9, 15, 20), implying their distinct functions in various cellular processes. For example, integrin ligands or antibodies bind to hemocytes and inhibit different cellular immune processes in *Drosophila melanogaster* (21), *Litopenaeus vannamei* (22), *Biomphalaria glabrata* (23), *Apostichopus japonicus* (24), and *Crassostrea gigas* (25). Besides, invertebrate integrins also participate in multiple cellular immune responses mainly mediated by human αI-domain containing integrins, although the αI-domain is evolutionarily absent in invertebrates (7, 26–28), which suggests the existence of certain complex functional compensatory mechanisms in invertebrates. Moreover, some invertebrate integrins mediate cellular immune responses, such as hemocyte encapsulation and melanization (29, 30) that occur to a limited extent in invertebrates, which indicates their functional diversity in invertebrate immunity. Obviously, invertebrate integrins effectively link multiple immune responses and exhibit complex and sophisticated functional divisions and cooperation during migration, phagocytosis, and other immune responses generated by immunocytes (3, 26), while few studies are focused on the invertebrate integrins with immune functions, or mainly limited in model invertebrates (7). A comprehensive understanding of integrin-mediated immune responses in other invertebrates existing in different environments is still needed, which will promote our understanding of the diversity and specificity of innate immunity in invertebrates.

To understand the functional mechanisms of integrins as cell receptors, it is important to identify their specific ligands, whereas the information is generally not available for invertebrates (31, 32). Human integrins, for example, can be classified into three types based on the binding target motifs. Leucine-aspartic acid-valine (LDV)-binding receptors bind to some laminin and collagen isoforms with LDV motifs (32, 33). Arginine-glycine-aspartate (RGD)-binding receptors and laminin-binding receptors recognize the RGD motif in native ligands, such as those occurring in fibronectins, vitronectins, fibrinogens, and laminin isoforms in the extracellular matrix (ECM) (32, 34–36). Collagen-binding receptors are αI-domain-containing integrins that recognize a specific motif, that is, glycine-phenylalanine-hydroxyproline-glycine-glutamate-arginine (GFOGER), derived from a subset of collagens (9, 37, 38). Because specific integrin family members bind to specific ligands, functional divisions are established for the highly evolved human integrin family, with regard to the ligand binding process (32). In invertebrates, emerging evidence suggests that invertebrate integrins have diversified special structures that provide the basis for binding multiple ligands (15, 21, 30, 37). However, only the binding of certain invertebrate integrins to RGD-containing proteins has been confirmed (23, 24), and the binding of these integrins to other typical ligands, including LDV-containing proteins, GFOGER-containing proteins, and laminins has not been reported. Because of the high sequence variability and uncertainty in the phylogenetic branches of invertebrate integrins, it is more possible to categorize them effectively based on their ligand-binding properties and to clarify the functional mechanisms that enable their categorization and cooperation.

Although information regarding the evolutionary and functional relationships among invertebrate integrins is not clear, and limited by the few genome-sequenced species, more and more integrin family members are identified in non-model invertebrates, such as in the Mollusca, which is the biggest phylum in the marine animal kingdom with the most species. The molecular aspects of some integrins in mollusks such as *Lymnaea stagnalis* (39), *Mytilus trossulus* (40, 41), *B. glabrata* (23, 42), *Mytilus galloprovincialis* (43), and *C. gigas* (25, 44–46) have been described, but the complete access to information regarding any of the mollusk species remains unavailable. The Pacific oyster (*C. gigas*), a sessile filter feeder exposed to a wide range of biotic and abiotic stresses, is an attractive model to study the diversity of immune receptor families and the mediation of associated immune responses in invertebrates (47, 48). The objectives of this study are (1) to identify all integrin family members from *C. gigas* genome, and to characterize their structural and evolutionary features; (2) to investigate their functional characteristics during the ligand binding process; and (3) to determine their mediation mechanism in different cellular immune responses, and to clarify their possible functional patterns. The results will provide systematic data to improve our understanding of the classification, structure, and evolutionary characteristics of the integrin family in a marine invertebrate animal and thus add to the evidence essential for the diversity and specificity of immune responses in invertebrates.

## Materials and Methods

### Searching, Screening, and Identifying *C. gigas* Integrin Family Members

Because the reported integrins in human and drosophila commonly contain conserved domains with two to five extracellular Intα domains (beta-propeller repeats) and an integrin_alpha2 (INA) in α integrins, or an extracellular integrin_beta (INB) (or called βI-domain) in β integrins, in addition to a transmembrane domain in both α and β integrins (32), oyster α/β integrins were screened from the entire genome, according to this criterion. As shown in the pipeline in Figure S1, the putative genes of α and β integrin were retrieved from the *C. gigas* genome (oyster_v9, http://ensemblgenomes.org) using HMMER3.1 software (49) with a multi-sequence alignment algorithm and with default parameters using the INA domain (PF08441) or INB domain (PF00362) as templates. Domain prediction analysis was performed to verify the putative integrin family members using the SMART program (http://smart.embl-heidelberg.de/). The presence/absence of the transmembrane region was evaluated by TMHMM program (http://www.cbs.dtu.dk/services/TMHMM/).

### Sequence Analysis

To compare and investigate the domain-related features of oyster integrins, the well-studied integrin family members, including five α and two β integrins from *D. melanogaster*, and eighteen α and eight β integrins from *H. sapiens* were retrieved from NCBI (http://www.ncbi.nlm.nih.gov). The information regarding integrins and GenBank accession numbers had been shown in Table S1. The obtained sequences were subjected to Protein BLAST, to predict their domain composition, and the results were generated by DOG 2.0 (50). Multiple sequence alignments were performed using ClustalX 1.81 software, and the results were generated in the online platform (http://www.bio-soft.net/sms/) (51). The MEGA 6.06 software was used with the neighbor-joining algorithm to construct phylogenetic trees, and the results were tested for reliability over 1,000 bootstrap replicates, after which the editing was carried out online using the iTOL tool (http://itol.embl.de/) (52).

### Animal Manipulation and Sample Collection

Five-week-old female Kunming mice were provided by the Qingdao Institute of Drug Control for preparing antibodies. Oysters 10–12 cm in length were collected from an aquaculture farm in Rongcheng, China, and cultured in a sea water tank for 2 weeks to acclimate prior to processing. All experiments involving animals reported in this study were approved by the Ethics Committee of the Institute of Oceanology, Chinese Academy of Sciences. For the immune challenge experiments, 48 oysters were divided into two groups. Twenty-four oysters stimulated by the injection with 100 μL of lipopolysaccharide (LPS) (1 mg/mL) after 12 h were included in the LPS stimulation group, and the other untreated oysters were used in the blank control group. Twenty individuals were randomly sampled from each group, and the hemolymph samples (1 mL per oyster) were collected from oyster hematococoel using an injection syringe. Afterwards, the hemocytes were pelleted from the hemolymph by centrifugation at 800 g for 10 min at 4°C. The hemocytes were transferred and resuspended in modified L15 medium (0.54 g/L KCl, 20.2 g/L NaCl, 0.6 g/L CaCl_2_, 3.9 g/L MgCl_2_, 1 g/L MgSO_4_) (53), after which the cellular immune responses were evaluated.

### RNA Extraction and Gene Cloning

Total RNA was extracted from oysters using the Trizol reagent (Invitrogen), and cDNA strands were synthesized for use as templates by M-MLV reverse transcriptase (Promega), according to the manufacturer's instructions. The partial cDNA sequences of oyster integrin genes (Table S2) were amplified using ExTaq DNA polymerase (Takara), and the primers used for cloning were listed in Table S3. The Polymerase Chain Reaction (PCR) products were inserted into the pMD19-T simple vector (Takara) and verified by nucleotide sequencing.

### Protein Recombination and Purification

The Polymerase Chain Reaction (PCR) products of partial cDNA sequences of oyster integrin genes, mainly the extracellular part of integrin protein, with 5′ BamHI and 3′ EcoRI restriction site, were integrated into the pET-30a expression vector (Novagen) and expressed in the *Escherichia coli* Rosetta (DE3) system (TransGen Biotech). Nickel affinity column chromatography was employed to purify recombinant His-tagged integrin protein fragments (54). The purified recombinant proteins were dialyzed against TBS (50 mM Tris-HCl, 150 mM NaCl, pH 7.4) thrice at 4°C, and their concentration was determined according to the BCA assay kit (Sigma-Aldrich).

### Polyclonal Antibody Preparation

Polyclonal antibodies were prepared as described previously (55). Briefly, recombinant His-tagged integrin protein fragments (1 μg/μL) were emulsified using Freund's complete adjuvant (Sigma-Aldrich) separately, and then used to immunize a 6-week-old female mouse. The second and third immunizations were performed on the 16th and 30th day with the incomplete adjuvant. The fourth inoculation was executed on the 37th day using purified proteins. Afterwards, the serum of the mouse was collected on the 44th day, and the mouse polyclonal antibody was purified from immune serum using an IgG Purification Kit-G (Dojindo), according to the manufacturer's protocol.

### Expression Data Analysis

The available reads per kilobase million (RPKM) values of integrin genes for the stages of tissue expression and development were obtained from the previous transcriptome data, released by the oyster genome project (47). Fragments per kilobase million (FPKM) values for the expression of oyster integrins were obtained against LPS stimulation from another set of RNA-seq data (56). The values were normalized via log_2_ conversion, after which heatmaps were drawn to display the expression patterns of integrin family genes. The α-β integrin pairings were predicted according to heatmap clusters of gene co-expression, based on Pearson correlation coefficients (57).

### The Detection of Ligand Binding Ability

Peptide ligands, including RGD-containing peptide (RGDCP), LDV-containing peptide (LDVCP), GFOGER-containing peptide (GFOGERCP), FITC labeled-RGDCP, LDVCP, and GFOGERCP with a purity of >95% were synthesized by Sangon Biotech (10 mg, Shanghai, China). The protein ligand of laminin was purchased from Abcam (Cambridge, England). All were stored at −20°C before use.

The binding activities of the recombinant integrins toward four ligands, including RGDCP, LDVCP, GFOGERCP, and laminin proteins were detected on a BIAcore T200 Surface Plasmon Resonance (SPR) instrument (GE Healthcare). The detailed setups of experimental instrument were performed as described with modifications (54). HBS-EP (GE Healthcare) (pH 7.5), which was used as the running buffer, contained 10 mM HEPES, 150 mM NaCl, 3 mM EDTA, and 0.005% (v/v) Surfactant P20. First, an anti-His-tag antibody was immobilized onto the CM5 sensor chip surface, according to the instructions in the Amine Coupling Kit (GE Healthcare). The recombinant integrin proteins with a His-tag (0.1 mg/mL) were injected and allowed to bind to the anti-His-tag antibody with approximately 200 response units (RUs). Three peptides of RGDCP, LDVCP, GFOGERCP, and laminin protein (0.1 mg/mL) were injected into the wells, and flow wells were controlled at a flow rate of 10 μL/min for 180 s. Finally, the bound proteins and ligands were washed for 30 s with 10 mM glycine-HCl (pH = 1.5), at a flow rate of 20 μL/min. The binding reaction was described as the relative reaction value that was equal to the highest reaction value minus the baseline reaction value, based on the raw results obtained using the SPR instrument. The binding reactions for which the RUs were >10 were considered as positive reactions (54). The binding reaction of recombinant thioredoxin (rTRX) to each kind of ligand was used as the negative control.

### Subcellular Localization Analysis and Ligand-Hemocyte Binding Assay

To detect the subcellular localization of integrin proteins, 20 μL of hemocytes (10^6^ cells/mL) in modified L15 medium was seeded onto positively charged glass slides for 30-min cell adhesion and fixed with 4% paraformaldehyde prepared in PBS for 15 min at room temperature, followed by blocking with 3% BSA solution (in PBS) for 1 h. After extensively washing the slides, the hemocytes were incubated with a mouse-anti integrin antibody solution at a dilution of 1:250 (in 3% BSA solution) for 1 h. The slides were washed with PBS, after which the hemocytes were incubated with the Alexa Fluor 594-conjugated goat anti-mouse secondary antibody (Abcam) solution at a dilution of 1:250 (in 3% BSA solution), for another 1 h. Finally, the nuclei were stained with 4′,6-diamidino-2-phenylindole (DAPI) for 5 min and observed under a laser confocal scanning microscope (Carl Zeiss LSM 710).

An immunofluorescence assay was performed to detect the binding activity of hemocytes to three FITC-labeled integrin ligands. First, the collected hemocytes were resuspended in modified L15 medium (10^6^ cells/mL) and separately incubated with FITC-labeled RGDCP, LDVCP, and GFOGERCP (0.01 mg/mL) for 30 min. After washing the hemocytes extensively with modified L15 medium, they were fixed with 4% paraformaldehyde on glass slides and stained with DAPI, followed by observation under a laser confocal scanning microscope (Carl Zeiss LSM 710). The percentage of hemocytes stained with FITC was detected by flow cytometry (BDFACSArial). To block the binding of hemocytes to FITC-labeled ligands, samples in the blocking group were incubated with four different mouse-anti integrin antibodies, including three antibodies against the α integrins (*Cg*α1~3) and one β integrin (*Cg*β1) (diluted by 1:100 in modified L15 medium). There were three replicates for each sample.

### Phagocytosis, Encapsulation, and Migration Assay

The phagocytosis assay was conducted according to the method described previously (58). Firstly, *E. coli* treated with 4% formaldehyde for 10 min was washed three times with 0.1 M NaHCO_3_ (pH 9.0), and then incubated with 1 mg/mL FITC (Sigma-Aldrich) overnight. After the washing of unbound FITC with PBS buffer for three times, the FITC-labeled bacteria were prepared for phagocytosis assay. Hemocytes (10^6^ cells, 1 mL) were incubated with FITC-labeled *E. coli* (10^8^ bacteria, 10 μL) at room temperature for 1 h to incite phagocytosis, and the unphagocytosed bacteria were washed by modified L15 medium for three times. The phagocytosis rate was finally detected by flow cytometry (BDFACSArial) (58). For the blocking group, the hemocytes were incubated with the four kinds of integrin ligands (0.01 mg/mL), including label-free RGDCP, LDVCP, GFOGERCP, and laminin protein or four kinds of mouse-anti integrin antibodies (diluted by 1:100 in modified L15 medium), including three antibodies against α integrin proteins (*Cg*α1~3) and one β integrin protein (*Cg*β1) for 1 h prior to the incubation with FITC-labeled *E. coli* for cell phagocytosis. There were three replicates for each sample.

The encapsulation assay was carried out according to the method described previously (59). Briefly, Ni-NTA agarose beads (Qiagen) were washed and resuspended in modified L15 medium at 100–120 beads/μL. Then, 1 μl of beads were incubated with 200 μl of hemocytes (2 × 10^6^ cells/mL). The mixture was added into a well in a 1% agarose-coated cell culture plate (Costar) and incubated at 18°C for 6 h. The encapsulation of the agarose beads was observed under a microscope after incubation, and encapsulation rates were calculated as described previously (59). For the blocking group, the hemocytes were incubated with integrin ligands or antibodies as in the phagocytosis assay, and then incubated with Ni-NTA agarose beads for encapsulation.

Cell migration was surveyed using EMD Millipore MultiScreen™ 96-well assay plates (Millipore, pore size: 5.0 μm), based on the method described previously (60). Briefly, the prepared hemocytes were washed twice with modified L15 medium and resuspended at a concentration of 5 × 10^6^ cells/mL. One microliter of 5 mM Calcein AM (Invitrogen) was added into the suspension to label the hemocytes for 30 min at room temperature. After washing the unbound Calcein AM, 50 μL of labeled hemocytes were mixed with 150 μL of modified L15 medium containing 10% fetal bovine serum (TransGen Biotech). The fluorescence of each well was measured to indicate the total number of hemocytes (Ex/Em = 494/517, Value 1). After 60 min, the undersides of inserts and the receiver were carefully washed and swabbed with modified L15 medium to remove all migrated hemocytes. Afterward, the fluorescence measurement of each well was repeated to indicate the total number of non-migrant cells (Value 2). The migration rate was calculated after all trials were conducted with three replicates (Migration rate% = 100% – Value 2/Value 1). Negative controls employed the hemocytes that were seeded into the wells in which FBS was omitted from the modified L15 medium. For the blocking group, the migration rates of hemocytes were determined after hemocytes were incubated with the four integrin ligands (0.01 mg/mL) or the four mouse-anti integrin antibodies (diluted by 1:100 in modified L15 medium) for 1 h.

### Statistical Analysis

Results were shown as mean ± S.D values. The two-sample Student's *t*-test was performed for the comparisons conducted between groups; ^*^stands for statistical significance (*p* < 0.05) and ^**^stands for extremely statistical significance (*p* < 0.01).

## Results

### Identification of Multiple Integrin Family Members From *C. gigas*

Integrin commonly functions as the heterodimer assembled by two distinct α and β integrins (14), while the complete information about integrin family including α and β integrins in any of the mollusk species remains unavailable. In order to screen the entire integrin family members, both α and β integrin genes were retrieved against the oyster genome based on HMMER 3.1 program. The results showed that there were nine α and twelve β integrin candidate genes in the oyster genome (Tables S4, S5). Among these candidates, the deduced eight α integrin proteins (CGI_10008246, CGI_10005638, CGI_10010727, CGI_10012356, CGI_10012568, CGI_10013155, CGI_10021391, and CGI_10023513) contained a transmembrane domain, 1–3 Intα domains, and an INA domain in their extracellular region, and they were identified as α integrins in *C. gigas* (Figure S2). α CGI_10017565 was an exception in that it had no transmembrane region (Figure S2). For oyster β integrins, three candidates (CGI_10012179, CGI_10012180, and CGI_10014761) were predicted with an intact intracellular cytoplasmic region, a transmembrane domain, and extracellular INB domain, and identified as β integrins in *C. gigas*, while the other nine candidates were excluded because they lacked transmembrane domains (Figure S3). As a result, the oyster integrin family consisted of eight α and three β integrins, which were named as *Cg*α1~8, and *Cg*β1~3, respectively, based on the E-values from low to high calculated by HMMER 3.1 program (Table 1). The number of integrin family genes in the oyster was less than that in the genomes of *H. sapiens* and the tunicate, *Ciona intestinalis*, but it was more than in *D. melanogaster, C. elegans*, and *N. vectensis* (Table 2). This indicated that the number of integrin family genes in the genome of *C. gigas* had expanded compared to evolutionarily higher organisms such as *D. melanogaster*.

**Table 1:** The integrin genes identified in *C. gigas* genome.

	**Gene ID**	**Gene name**	**Accession number**	**Scaffold**	**Position**	**Full length (bp)**	***E*-value**
α	CGI_10013155	*Cg*α1	EKC18351	43844	247645–278826	2622	5e-81
	CGI_10012356	*Cg*α2	EKC18458	43786	234476–275282	2076	2.9e-76
	CGI_10021391	*Cg*α3	EKC41508	115	394752–406446	1644	4.8e-29
	CGI_10023513	*Cg*α4	EKC28745	1258	789902–820003	3114	1.3e-27
	CGI_10010727	*Cg*α5	EKC18839	43530	25078–54104	3087	9.1e-22
	CGI_10008246	*Cg*α6	EKC29903	1148	57234–80106	3003	2.2e-12
	CGI_10012568	*Cg*α7	EKC38514	336	195464–211672	3237	1.1e-10
	CGI_10005638	*Cg*α8	EKC20678	41792	72647–92865	4026	2.2e-4
β	CGI_10012179	*Cg*β1	EKC18490	43764	121281–149056	2430	8.2e-109
	CGI_10014761	*Cg*β2	EKC35462	542	109727–126005	2058	2.8e-71
	CGI_10012180	*Cg*β3	EKC18491	43764	153863–169907	1758	1.4e-52

**Table 2:** The expanded oyster integrin family members compared to other species.

	***H. sapiens***	***C. intestinalis***	***D. melanogaster***	***C. gigas***	***C. elegans***	***N. vectensis***
α	18	11	5	8	3	3
β	8	5	2	3	1	4

*The numbers of integrin gene family members from the genomes of different evolutionary ladder species, including mammalian H. sapiens, Tunicata C. intestinalis, Arthropoda D. melanogaster, Mollusca C. gigas, Nematoda C. elegans, and Cnidaria N. vectensis, are compared*.

### The Variable Sequence and Structure of Oyster α/β Integrins

**Figure 1:**
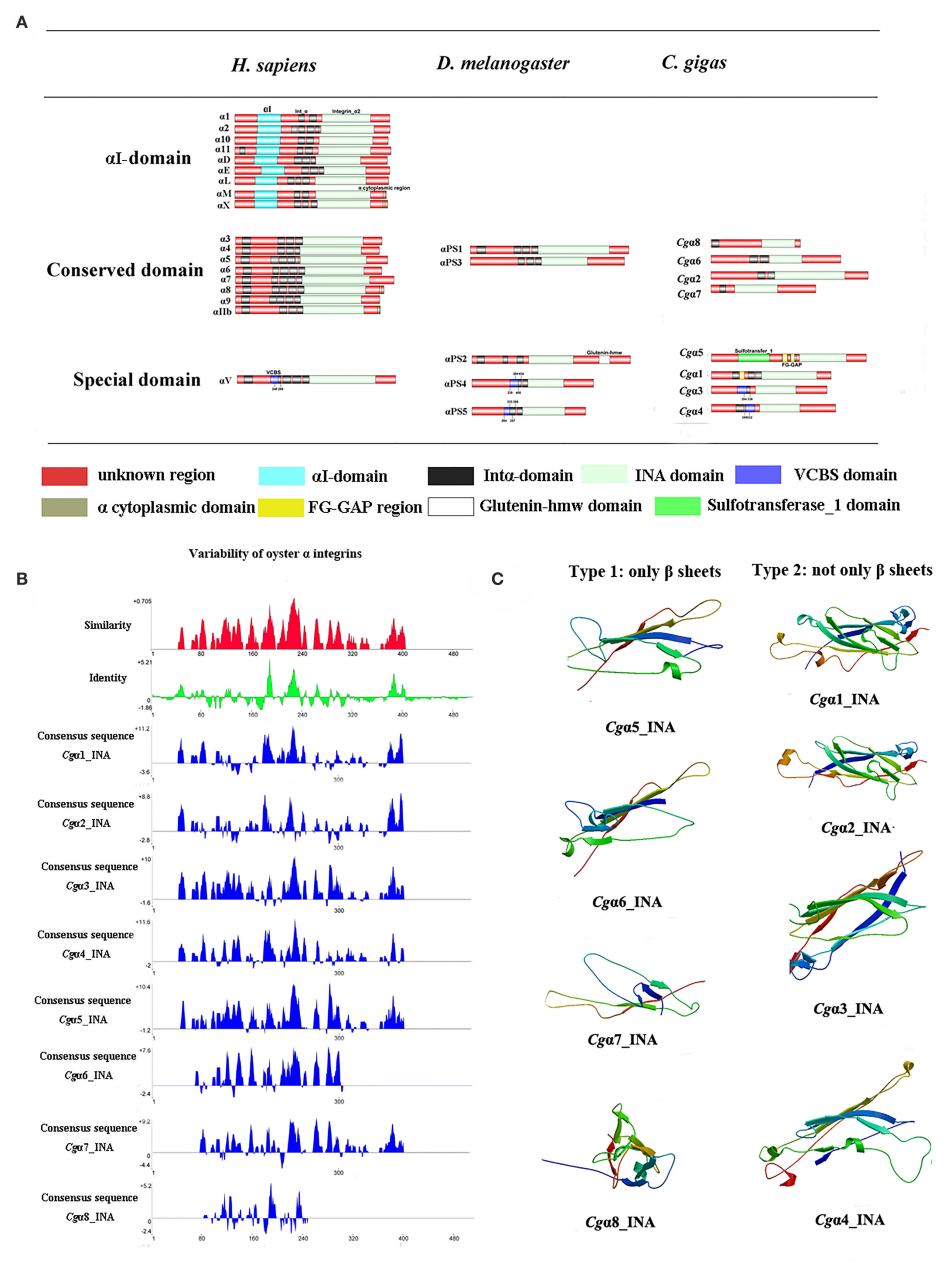
The features of domain composition and structure of oyster α integrins: **(A)** comparison of domain composition from *H. sapiens, D. melanogaster*, and *C. gigas* α integrins; **(B)** identity and similarity of INA domains in oyster α integrins. The sequence alignment analysis is conducted by VectorNTI 10 software to reveal the conserved and similar amino acid sites among oyster INA domains. The red and green histograms show the overall similar and conserved amino acid sites of INA domains in oyster α integrins, and the blue histograms show the conserved amino acid sites of each INA domain of oyster α integrin; and **(C)** three-dimensional predicted ribbon structures of INA domains in oyster α integrins by SWISS-MODEL (https://swissmodel.expasy.org/).

**Figure 2:**
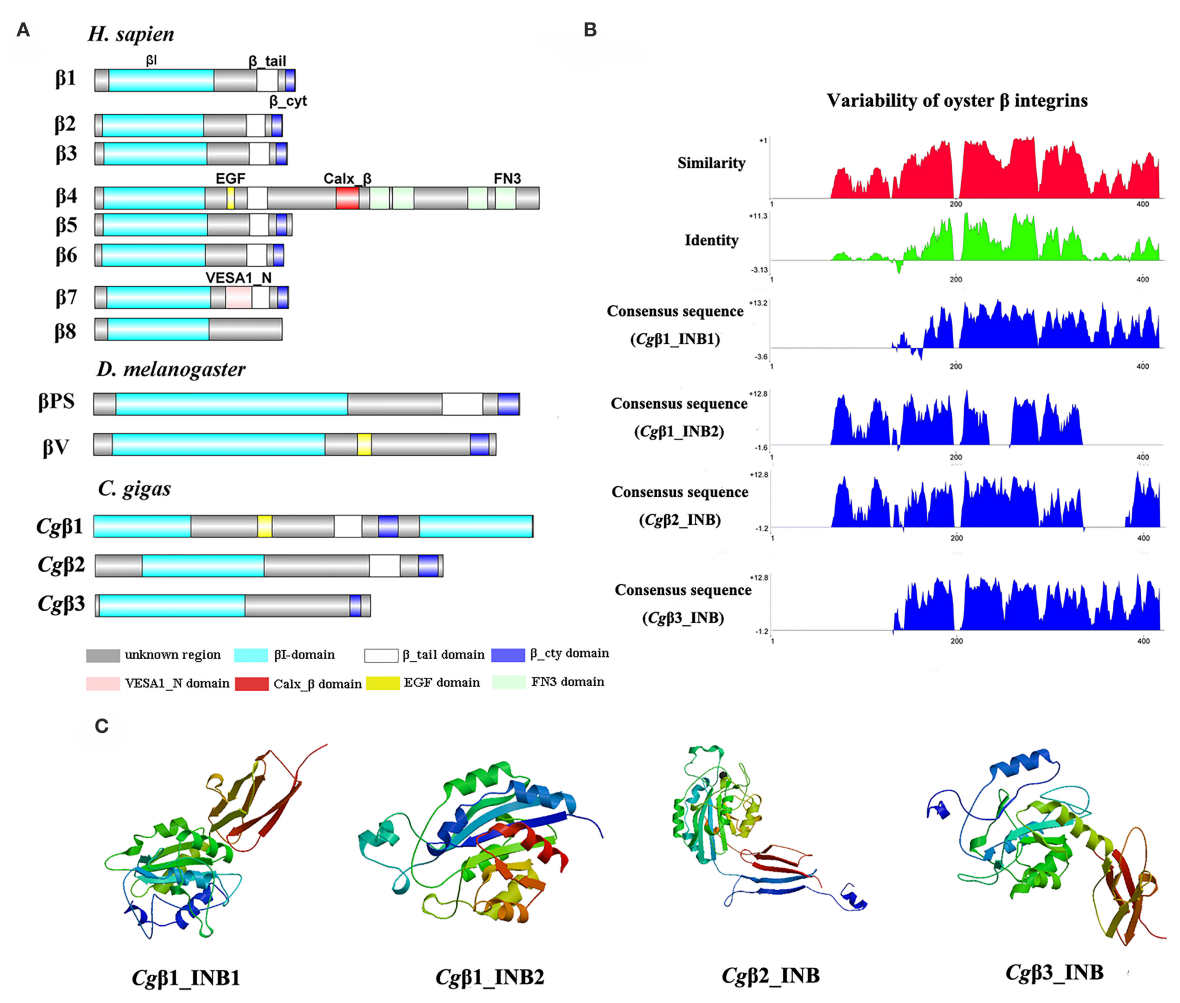
The features of domain composition and structure of oyster β integrins: **(A)** comparison of domain composition from *H. sapiens, D. melanogaster*, and *C. gigas* β integrins; **(B)** identity and similarity of INB domains in oyster β integrins. The sequence alignment analysis is conducted by VectorNTI 10 software to reveal the conserved and similar amino acid sites among oyster INB domains. The red and green histograms show the overall similar and conserved amino acid sites of INB domains in oyster α integrins, and the blue histograms show the conserved amino acid sites of each INB domain of oyster β integrin; and **(C)** three-dimensional predicted ribbon structures of INB domains in oyster β integrins by SWISS-MODEL (https://swissmodel.expasy.org/). INB domain was alternatively called βI-domain in this study.

Because the information about the structure for invertebrate integrins is still limited, the features of oyster integrin structure were comprehensively surveyed. Because of the great structural difference between α and β integrins, the structural features of oyster α/β integrins were separately analyzed. The domain composition of α integrin proteins from human, drosophila, and oyster was compared in Figure 1A. The results revealed that neither oyster nor drosophila α integrin proteins had αI-domains, which were present only in nine human α integrin proteins. Other more noteworthy domains were identified in oyster α integrin proteins than the corresponding proteins in human and drosophila (Figure 1A). Specifically, *Cgα*3 and *Cgα*4 contained an extracellular VCBS domain that was similar to that in human αV and drosophila αPS4 and αPS5, while *Cgα*5 and *Cgα*1 had extracellular FG-GAP regions, and *Cgα*5 contained a sulfotransferase domain. The latter two domains were not observed in human and drosophila α integrins (Figure 1A). For the comparison about the domain composition of β integrins from human, drosophila, and oyster, Figure 2A shows that all β integrin proteins harbor the hallmark INB domain in their extracellular regions. An exception was *Cgβ*1, which had two INB domains (designated as INB1 and INB2) in the extracellular and intracellular regions, respectively (Figure 2A). This indicated that some of oyster α/β integrin proteins owned several distinct domain components compared to those in human and drosophila.

Because INA and INB domains are the essential parts of integrin (31, 37), their sequence features in oyster integrins were analyzed in detail. The results showed that although the hallmark structures including INA and INB domains were observed in oyster α and β integrins, respectively, they exhibited different sequence conservation. WebLogo analysis revealed that only three amino acid sites, C^221^, C^228^, and L^232^, were conserved in INA domains of eight oyster α integrin proteins (Figure S4). The values for the consensus and identity positions were 23.8 and 0.2%, respectively, which were significantly lower than 25.3 and 1.7% for human integrin INA domains (Figure 1B and Figure S5). The sequence alignment of oyster integrin INA domains identified regions in which insertions and deletions of amino acids were leading to gaps in >50% of the aligned sequences (Figure 1B and Figure S5). On the other hand, multiple amino acid alignments of full-length amino acids showed that the GFFXR motif was present in the C-terminus for all 18 human α integrin proteins (Figure S6). In oysters, all α integrin proteins except *Cg*α7 also harbored this conserved GFFXR in the C-terminus (Figure S6), which indicated that some key amino acid sites for oyster α integrin proteins were conserved, even though they displayed a great degree of sequence variability. While the amino acid sequence alignment of oyster INB domains showed that consensus and identity positions accounted for approximately 67.1, and 14.4%, respectively (Figure 2B), which were very close to those of 69.6 and 14.9% in human integrin INB domains (Figure S7). The amino acids in positions 185–195, 209–233, and 261–285 in the alignment showed conservation of at least 50% of the sequences (Figure 2B and Figures S7, S8). Multiple sequence alignments of full-length amino acids showed that human β1, 2, 3, 5, 6, and 7 contained the NPXY/F motif in the C-terminus, which was also observed in the C-terminus of β integrins *Cg*β1 and *Cg*β2 in the oyster, although *Cg*β3 did not harbor this conserved motif (Figure S9). These results indicated that both the amino acid sequences of INA and INB domains from oyster integrin proteins exhibited variability, but more conserved amino acid sites were found in INB domains than in INA domains.

As the lengths of INA (~146–499 aa) and INB (~225–354 aa) domains from oyster integrins were much more varied and shorter than human integrins (322–500 aa for human INA domains, 416–446 aa for human INB domains; Figures S5, S7), the structural features of oyster INA and INB domains were further analyzed by SWISS-MODEL (61) using human INA (PDB: 3v4v) (62) and human INB (PDB: 4wk4) (63) as the templates (Figures 1C, 2C). Generally, INA domains are composed of three immunoglobulin-like domains with the conserved thigh and calf regions (9, 31). Our results showed that oyster INA domains had no conserved thigh and calf regions but had different numbers of β sheets and α helices, and thus formed notably different spatial structures (Figure 2C). Based on their structural composition, oyster INA domains could be simply classified into two types, including the INA domains with only β sheets (*Cg*α5~8) and the other type containing both α helices and β sheets (*Cg*α1~4). The tridimensional structure of INB domains showed that the conserved amino acid sequences appeared to be mainly located in the β sheet regions and revealed a remarkable conservation of the central structure with four to six β sheets that were surrounded by six to eight α helices. The modeled tridimensional structure of *Cg*β1_INB2 formed the only conserved central structure of the INB domain, while *Cg*β1_INB1 and *Cg*β3_INB contained six β sheets in their N-terminus, and *Cg*β2_INB contained five β sheets and one α helix in the C-terminus, in addition to the conserved central structure of the INB domain (Figure 2C). The results from the tridimensional structure analysis together with the sequence alignments indicated that the structures of oyster integrins were highly varied, and INA domains were much more variable than INB domains.

### Oyster α/β Integrins Were Clustered Into Species-Specific Phylogenetic Branches

**Figure 3:**
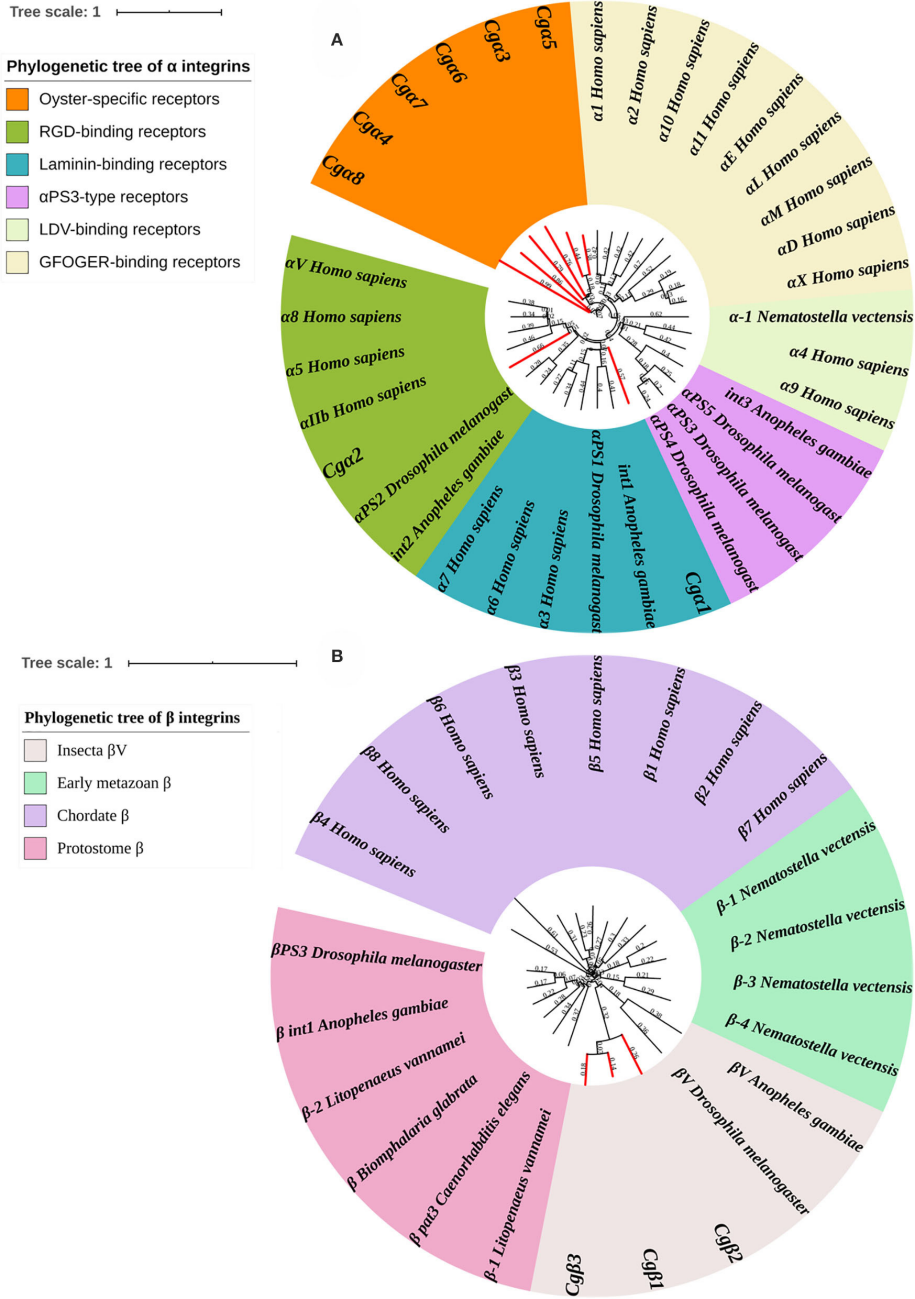
Phylogenetic analyses of α **(A)** and β **(B)** integrins to reveal the evolutionary characteristics of oyster integrins. Species, proteins, and Genbank accession numbers of integrins used in phylogenetic reconstructions are listed in Table S1. The branches for oyster integrins are marked red in phylogenetic trees.

Because α and β integrins have separate evolutionary histories, the phylogenetic analyses of α and β integrin protein sequences were usually conducted separately (7). For α integrins, they were usually reported as the key functional units for integrin ligand specificity and are further classified into LDV-binding receptors, RGD-binding receptors, GFOGER-binding receptors, laminin-binding receptors, and PS3-type receptors (32). In the present study, a phylogenetic tree with 35 α integrins selected from different species was constructed using the neighbor-joining method, and all members were distinctly separated into six distinct branches, including RGD-binding receptors, laminin-binding receptors, PS3-type receptors, LDV-binding receptors, αI-domain receptors, and oyster-specific receptors (Figure 3A). Although *Cgα*2 was clustered within the RGD-binding receptor branch, and *Cgα*1 was clustered within the laminin-binding receptor branch, α integrins of LDV-binding receptors, PS3-type receptors, and αI-domain receptors were absent in oysters, and the other six α integrins were all clustered into the oyster-specific α integrin branch (Figure 3A). β integrins exhibit more obvious phylum-lineage features, and when 23 β-integrins were evaluated by phylogenetics, they were segregated into four branches, including chordate β, protostome β, insecta βV, and early metazoan β, just as previously reported (9). Three oyster β integrins (*Cgβ*1~3) were clustered with the insecta βV and were distantly related to the β integrin from the mollusk species *B. glabrata* (Figure 3B). These results indicated that both oyster α and β integrins had been clustered into species-specific phylogenetic branches.

### Oyster α/β Integrins Were Spatiotemporally Expressed and Well Cooperative

**Figure 4:**
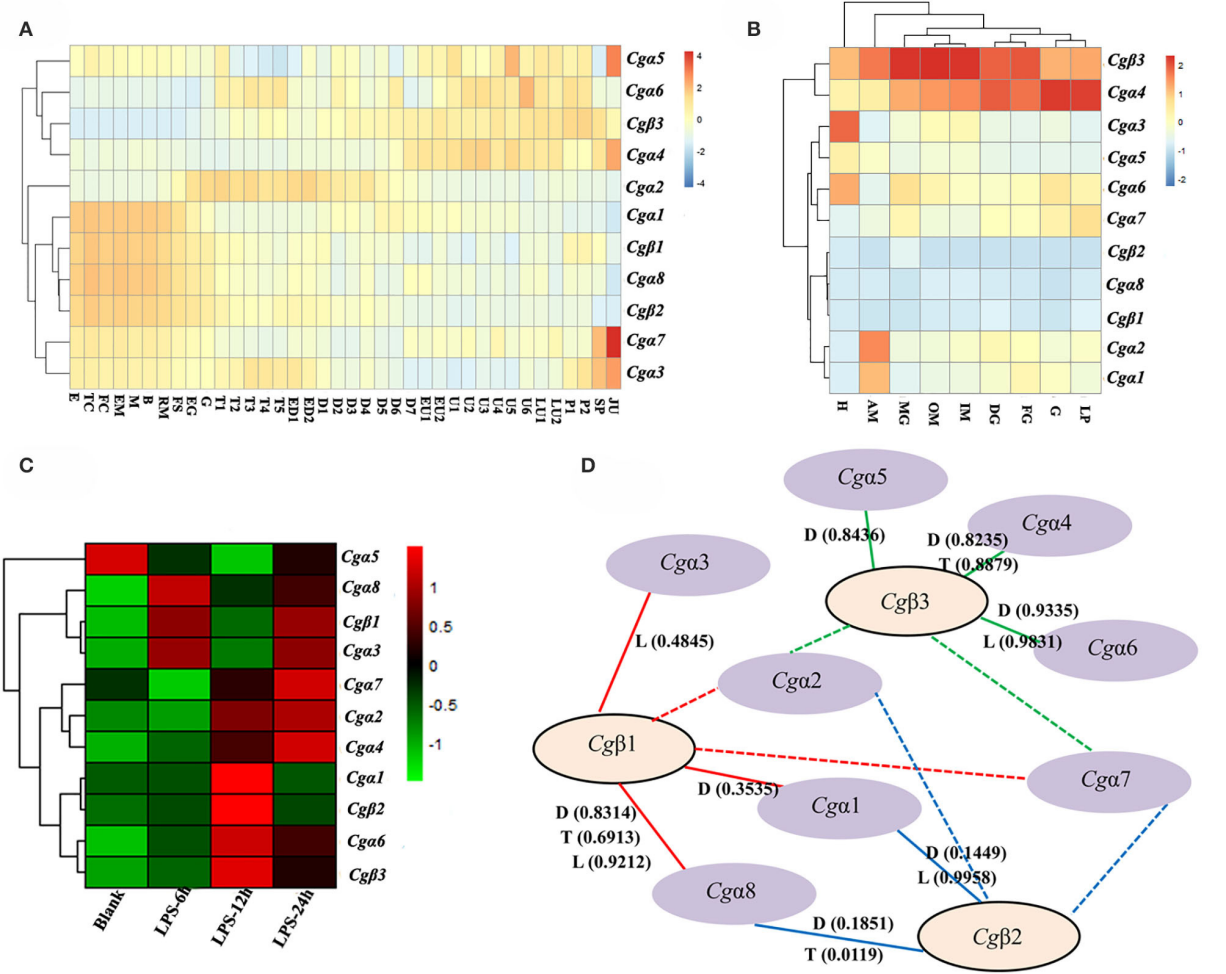
RNA-seq data set analysis to reveal the co-expression clusters of oyster α and β integrin genes in different developmental stages **(A)**, tissues **(B)**, time points in response to LPS stimulation **(C)**, and to predict potential α-β integrin heterodimers **(D)**. **(A)** Heatmaps indicate the gene expression level of integrin gene at different developmental stage. Developmental stages: E, egg; TC, two cells; FC, four cells; EM, early morula; M, morula; B, blastula; RM, rotary movement; FS, free swimming; EG, early gastrula stage; G, gastrula; T1, trochophore 1; T2, trochophore 2; T3, trochophore 3; T4, trochophore 4; T5, trochophore 5; ED1, early D-larva 1; ED2, early D-larva 2; D1, D-larva 1; D2, D-larva 2; D3, D-larva 3; D4, D-larva 4; D5, D-larva 5; D6, D-larva 6; D7, D-larva 7; EU1, early umbo larva 1; EU2, early umbo larva 2; U1, umbo larva 1; U2, umbo larva 2; U3, umbo larva 3; U4, umbo larva 4; U5, umbo larva 5; U6, umbo larva 6; LU1, later umbo larva 1; LU2, later umbo larva 2; P1, pediveliger 1; P2, pediveliger 2; SP, spat; and JU, juvenile; **(B)** heatmaps indicate the gene expression level of integrin gene in different tissues. Tissues: H, hemocytes; AM, adductor muscle; MG, male gonad; OM, outer mantle; IM, inner mantle; DG, digestive glands; FG, female gonad; G, gill; LP, labial palp; **(C)** heatmaps indicate the gene expression level of integrin gene at 0, 6, 12, and 24 h post-LPS stimulation; and **(D)** α-β integrin pairings predicted by the co-expression clusters in heatmaps based on Pearson correlation coefficients. The pairings supported by this analysis are indicated by continuous lines, and the unknown and possible pairings are indicated by dash lines. RNA-seq data are derived from developmental **(D)** stages, tissue (T) distribution, and LPS (L) stimulation. The positive Pearson correlation coefficients to predict α-β integrin pairings are shown in brackets and also listed in Table S6.

In order to explore the expression patters of oyster α/β integrin genes, three published transcriptome data sets for oyster development, tissue distribution, and the response to LPS stimulation, were analyzed. The results showed that oyster integrin genes were expressed during almost the whole early developmental stages (Figure 4A). Among them, two α integrin genes (*Cgα*1 and *Cgα*8) and two β integrin genes (*Cgβ*1 and *Cgβ*2) as one subgroup of genes were highly expressed before gastrulation (Figure 4A). The second group of one β integrin gene (*Cgβ*3) and three α integrin genes (*Cgα*4~6) were expressed after the D7 developmental stage (Figure 4A). *Cgα*2 was highly expressed from the developmental stage of G to D7, and two α integrin genes (*Cgα*3 and *Cgα*7) were highly expressed at the early and late developmental stages, which formed another two distinct subgroups (Figure 4A). These results suggested that oyster integrin genes were controlled and regulated with regard to the developmental stage to function at an appropriate time during early development.

For adult oysters, the expression patterns of oyster integrin genes in different tissues could be grouped into five subgroups (Figure 4B). *Cgβ*3 and *Cgα*4 showed relatively high expression levels in all tissues, whereas *Cgα*8 and two β integrin genes (*Cgβ*1 and *Cgβ*2) had relatively low expression levels in all tissues (Figure 4B). Three groups of tissue-specific integrins were also identified, including *Cgα*3 and *Cgα*5 that were highly expressed in hemocytes, *Cgα*6 and *Cgα*7 that were highly expressed in gonads, digestive glands, gills, and labial palps, and *Cgα*1 and *Cgα*2 that were highly expressed in the adductor muscle (Figure 4B). In addition, five integrin genes (*Cgα*3~6 and *Cgβ*3) had expression levels of >100 RPKM in hemocytes, whereas these genes showed expression levels of <100 RPKM in other tissues (Figure 4B), which indicated the specific and high expression of oyster integrin genes that occurred in hemocytes.

To further analyze oyster integrin gene expression in hemocytes in response to immune challenge, the expression was evaluated after LPS stimulation post 6, 12, and 24 h. Ten oyster integrin genes were up-regulated at different time points (Figure 4C). *Cgα*1 and *Cgβ*2 were up-regulated 12 h after LPS stimulation (Figure 4C). *Cgα*6 and *Cgβ*3 were up-regulated post 12 and 24 h after LPS stimulation (Figure 4C). Two α integrin genes (*Cgα*3 and *Cgα*8) and *Cgβ*1 were up-regulated 6 and 24 h after LPS stimulation (Figure 4C). The responses to LPS stimulation suggested the involvements of most oyster integrin genes in immune functions mediated by hemocytes.

Because all integrins function as α-β heterodimers and the paired α and β integrin genes in the same tissues usually exhibit co-expression during stress or normal development, the clusterings of oyster α/β integrin co-expression in heatmaps were analyzed based on Pearson correlation coefficients (Figure 4D). All the positive Pearson correlation coefficients were predicted to imply the high possibility of the clusterings of α/β integrin (Figure 4D and Table S6). Specifically, *Cgβ*1 might co-express with *Cgα*1, *Cgα*3, and *Cgα*8 (the red continuous lines), and *Cgβ*2 might co-express with *Cgα*1 and *Cgα*8 (the blue continuous lines), while *Cgβ*3 might co-express with *Cgα*4, *Cgα*5, and *Cgα*6 (the green continuous lines). The pairing pattern indicated a complex coordination relationship between oyster α and β integrins in different tissues or development stages during their mediation to various responses (Figure 4D).

### Oyster α/β Integrins Bound to Multiple ECM Ligands

**Figure 5:**
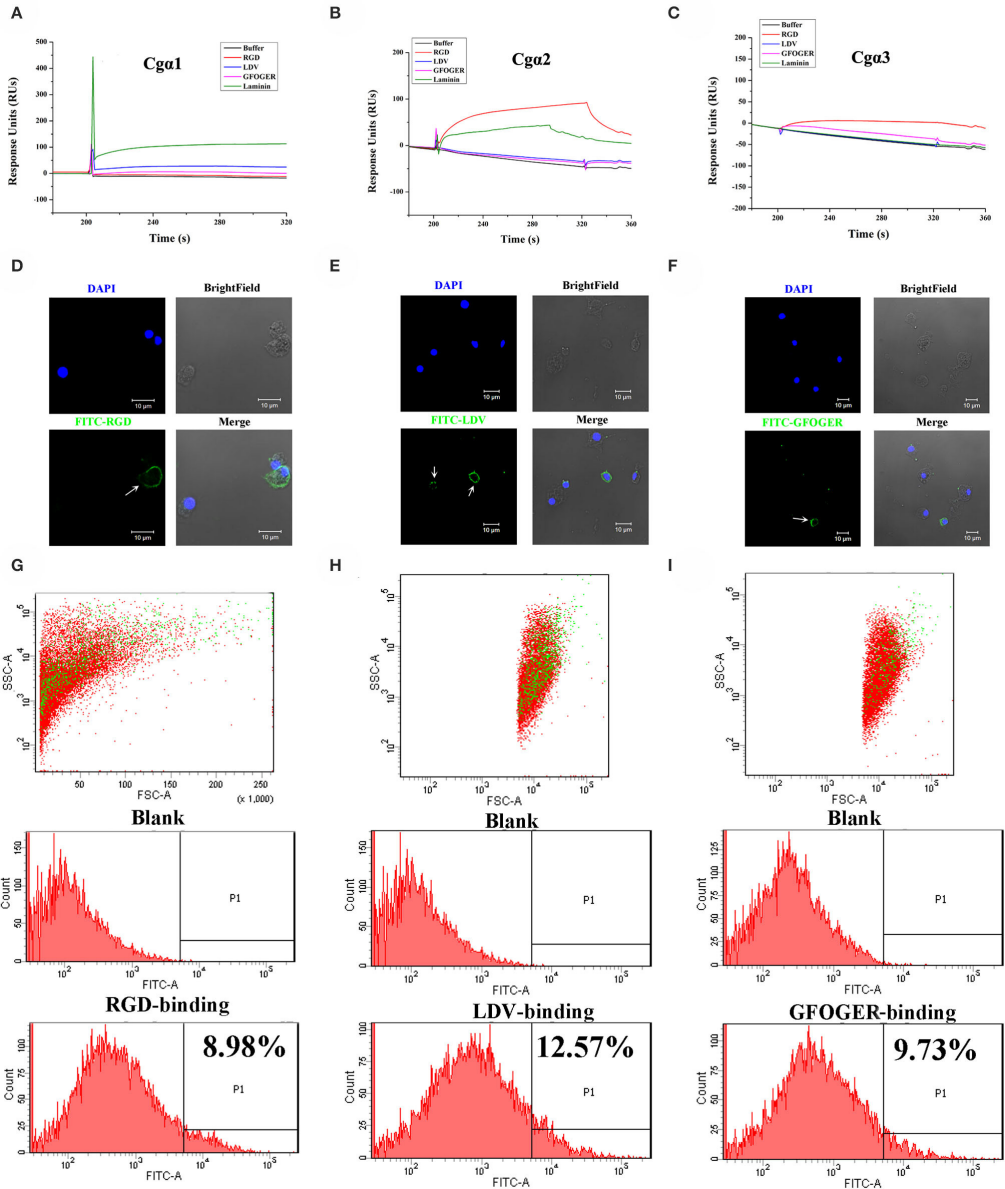
Oyster integrins bind to multiple ECM ligands. The binding reaction curves of recombinant protein fragments of *Cg*α1 **(A)**, *Cg*α2 **(B)**, and *Cg*α3 **(C)** to different ligands as analyzed by the SPR technique. The binding reactions for which the RUs >10 are considered as positive. rTRX is used as the negative control, and the binding reactions of rTRX to RGDCP, LDVCP, GFOGERCP, and laminin protein are <10 RUs (Figure S10). Immunofluorescence assays shows the oyster hemocytes binding to FITC-labeled RGDCP **(D)**, FITC-labeled LDVCP **(E)**, and FITC-labeled GFOGERCP **(F)**. The green fluorescence is derived from FITC-labeled peptides, and the blue fluorescence shows the DAPI-stained hemocyte nucleus. Flow cytometry analysis shows the percentage of hemocytes that bind to FITC-labeled RGDCP **(G)**, FITC-labeled LDVCP **(H)**, and FITC-labeled GFOGERCP **(I)**. The red and green dots stand for the whole obtained hemocytes and FITC-positive hemocytes analyzed by flow cytometry.

As the extracellular domain of integrin determines the ligand-binding activity (31), the fragment containing the key extracellular domains of 11 oyster integrin proteins was, respectively, recombined (Table S2), and then the interactions between the recombinant proteins and the ligands including RGD-containing polypeptide (RGDCP), LDV-containing polypeptide (LDVCP), GFOGER-containing polypeptide (GFOGERCP), and the laminin protein were analyzed by the SPR technique. The results showed that although eight recombinant integrin proteins failed to bind to any of the ligands (data not shown), the remaining three recombinants exhibited different ligand-binding abilities. Notably, *Cgα*2 bound to the laminin protein and the synthetic RGDCP (Figure 5A), and *Cgα*1 bound to the laminin protein (Figure 5B), while *Cgα*3 bound to both synthetic GFOGERCP and RGDCP (Figure 5C). The results displayed that oyster integrins could bind to typical ECM ligands, such as RGDCP, GFOGERCP, and laminin protein at the molecular level.

**Figure 6:**
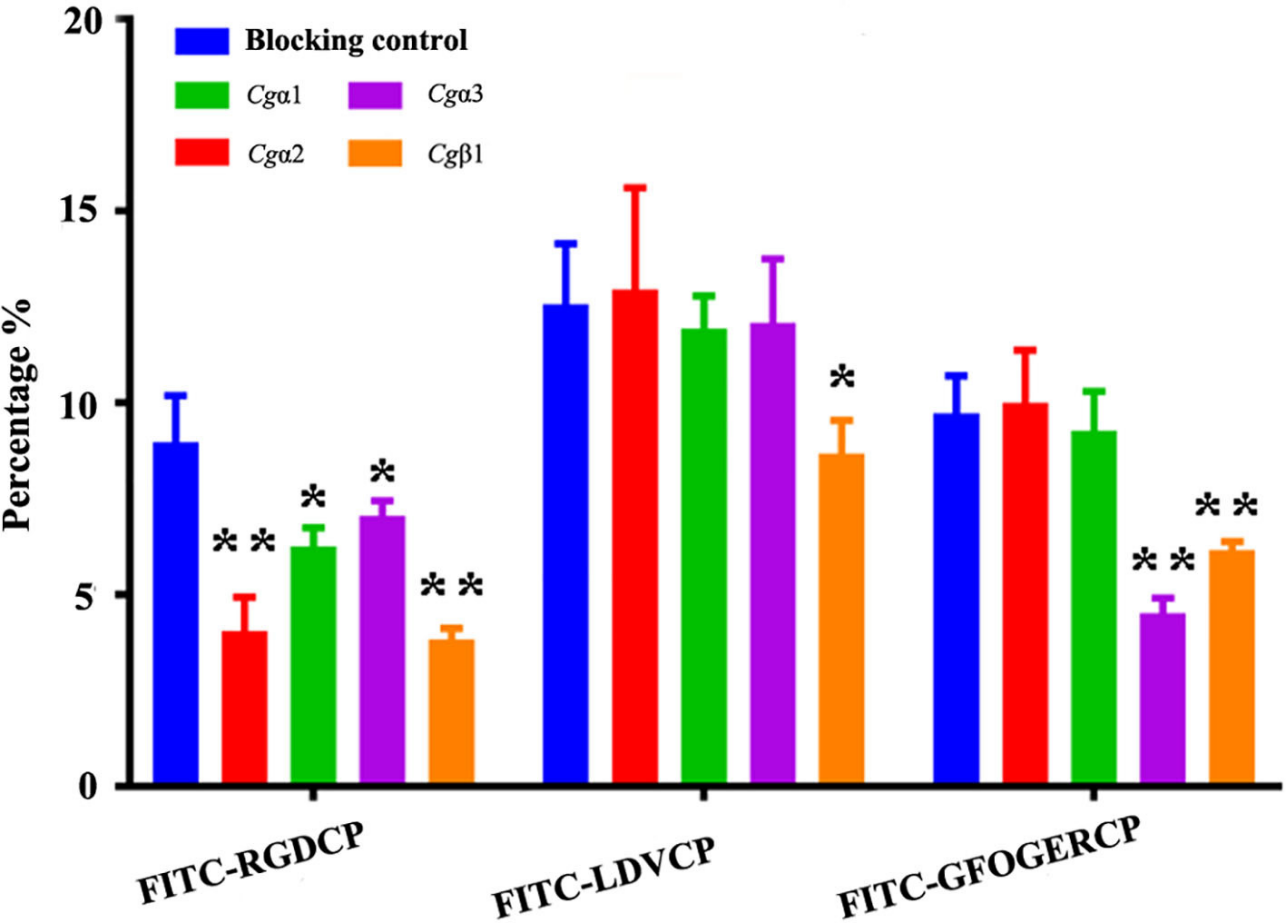
Oyster integrin antibodies inhibit the binding of integrin ligands to hemocytes. The percentage changes of FITC-labeled RGDCP positive hemocytes, LDVCP-positive hemocytes, and GFOGERCP-positive hemocytes are analyzed by flow cytometry after the hemocytes are blocked by the different antibodies against oyster integrin proteins including *Cg*α1~3, and *Cg*β1. Results are shown as mean ± SD (*n* = 3). *Stands for statistical significance (*p* < 0.05) and **stands for extremely statistical significance (*p* < 0.01), compared to the blocking control.

The binding activities of oyster integrins to different ECM ligands were also investigated at the cellular level. FITC-labeled RGDCP, LDVCP, and GFOGERCP were used as specific probes, and the results from confocal scanning microscopy showed that some of the hemocytes selectively bound to the three FITC-labeled peptides (Figures 5D–F). Further analysis by flow cytometry revealed that 8.98% of the hemocytes bound FITC-labeled RGDCP, 12.57% bound FITC-labeled LDVCP, and 9.73% bound FITC-labeled GFOGERCP (Figures 5G–I), which suggested that the integrin-located oyster hemocytes could selectively recognize and bind to multiple ECM ligands. To testify this observation, integrin representatives such as laminin-binding receptor *Cgα*1, RGD-binding receptor *Cgα*2, oyster-specific α *Cgα*3, and double INB domain containing *Cgβ*1 were selected based on the results of phylogenetic trees to further evaluate their ligand-binding properties by the antibody blocking assay (Figure 6). After the hemocytes were preincubated with the antibodies, the percentage of hemocytes that bound the FITC-labeled RGDCP were decreased from 8.98% in blocking control to 6.25% in *Cgα*1 group (*p* < 0.05), 7.06% in *Cgα*2 group (*p* < 0.05), 4.04% in *Cgα*3 group (*p* < 0.01), and 3.83% in *Cgβ*1 group (*p* < 0.01). Meanwhile, the antibodies to *Cgα*3 and *Cgβ*1 reduced significantly the percentage of hemocytes that bound the FITC-labeled GFOGERCP from 9.73% in blocking control to 4.52% in *Cgα*3 group (*p* < 0.01) and 6.17% in *Cgβ*1 group (*p* < 0.01). In addition, the percentage of hemocytes that bound to FITC-labeled LDVCP were also significantly reduced after the blocking of *Cgβ*1 antibodies, compared to the percentage bound to the blocking control (8.68 vs. 12.57%, *p* < 0.05). Collectively, the integrin ligand-binding activities of hemocytes were significantly inhibited by specific integrin antibodies, which confirmed the binding abilities of oyster integrins to multiple ECM ligands at the cellular level.

### Oyster α/β Integrins Located on the Surface of Different Hemocyte Subpopulations

**Figure 7:**
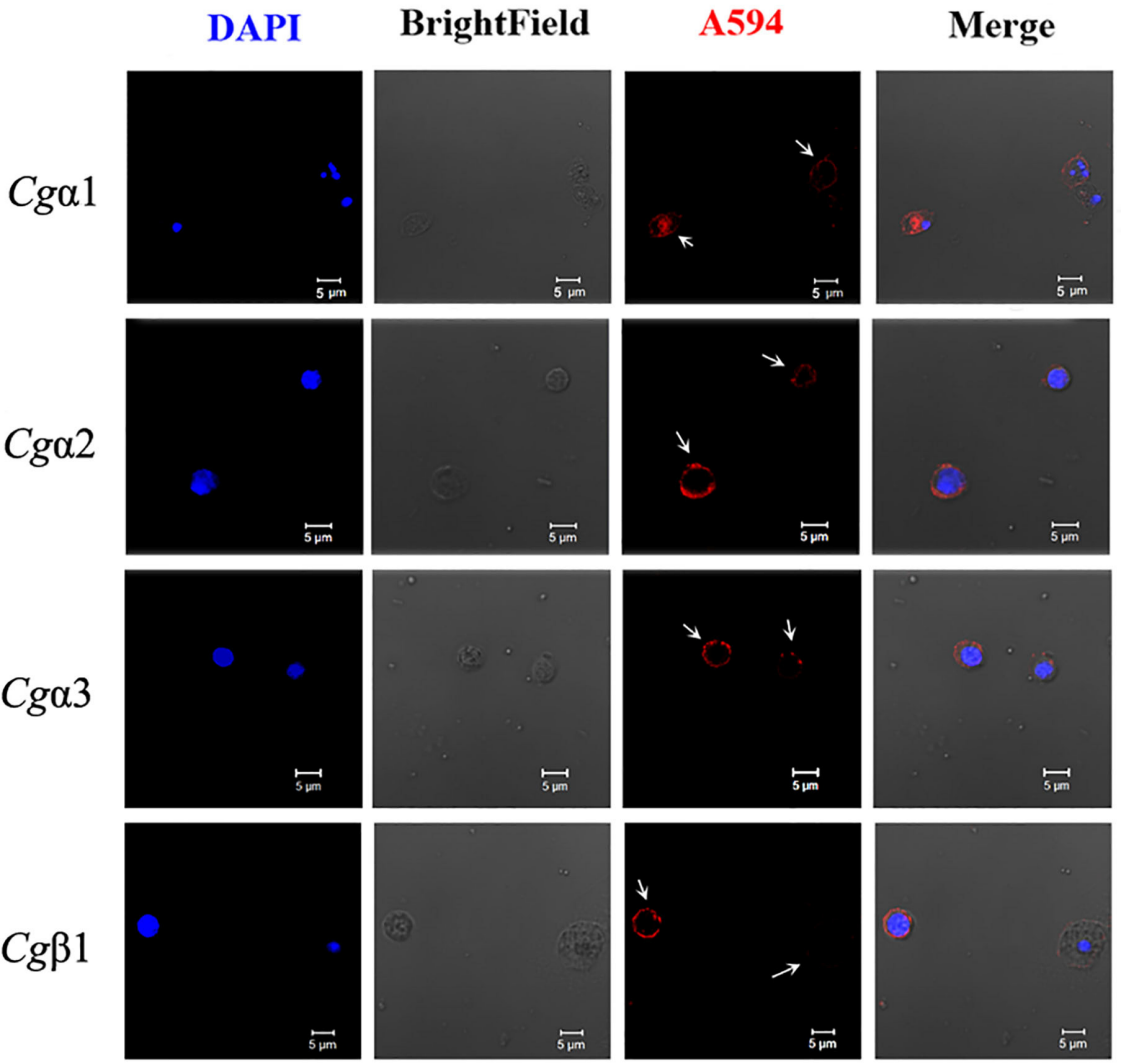
Different oyster integrins are distributed on the surface of specific hemocyte subpopulations. Subcellular localization of four oyster representative integrins, including *Cg*α1~3 and *Cg*β1, are analyzed by immunofluorescence. Oyster integrins are indicated by four mouse anti-integrin antibodies and Alexa Fluor 594-conjugated goat anti-mouse secondary antibody (red), and the hemocyte nucleus is stained by DAPI (blue). Bar = 5 μm. The white arrows are used to indicate the signals of Alexa Fluor 594.

The hemocytes that bound FITC-labeled RGDCP, LDVCP, and GFOGERCP were identified by different gates for the analysis by FSC-A and SSC-A in flow cytometry (Figures 5G–I). Hence, RGDCP-, LDVCP-, and GFOGERCP-binding integrins appeared to locate on different subpopulations of oyster hemocytes. It has been confirmed that oyster hemocytes with different size and cytoplasmic-nucleo ratio exert different major functions (48). In order to investigate the distribution characteristics of different oyster integrin family members on hemocytes, a further immunofluorescence assay with integrin antibodies was conducted (Figure 7). Notably, antibodies to *Cgα*2 and *Cgα*3 bound to the hemocyte subpopulation with a smaller cell size (5–10 μm) and a smaller cytoplasm to nucleus ratio relative to the other hemocyte subpopulations (Figure 7). The antibody to *Cgα*1 could bind to the both hemocyte subpopulations with a smaller and a larger cytoplasmic-nucleo ratio (Figure 7). The antibody to *Cgβ*1 also bound to the both hemocyte subpopulations with a smaller and a larger cytoplasmic-nucleo ratio, but the fluorescence was significantly brighter on the hemocyte subpopulation with a smaller cytoplasmic-nucleo ratio than that with a larger cytoplasmic-nucleo ratio, according to the red fluorescence intensity analysis (Figure 7). It seemed that the oyster integrin members were selectively distributed on the surface of hemocyte subpopulations with different size and cytoplasmic-nucleo ratio.

### Oyster α/β Integrins Were Involved in Multiple Cellular Defense Processes

In order to investigate the immune responses mediated by different oyster integrins, the cellular immune reactions including hemocyte phagocytosis, migration, and encapsulation by preincubating oyster hemocytes that were LPS-activated or not with four integrin ligands (RGDCP, LDVCP, GFOGERCP, and laminin proteins) and four integrin antibodies (*Cgβ*1, *Cgα*1~3) were detected, and the results were summarized as following.

#### Hemocyte Phagocytosis

**Figure 8:**
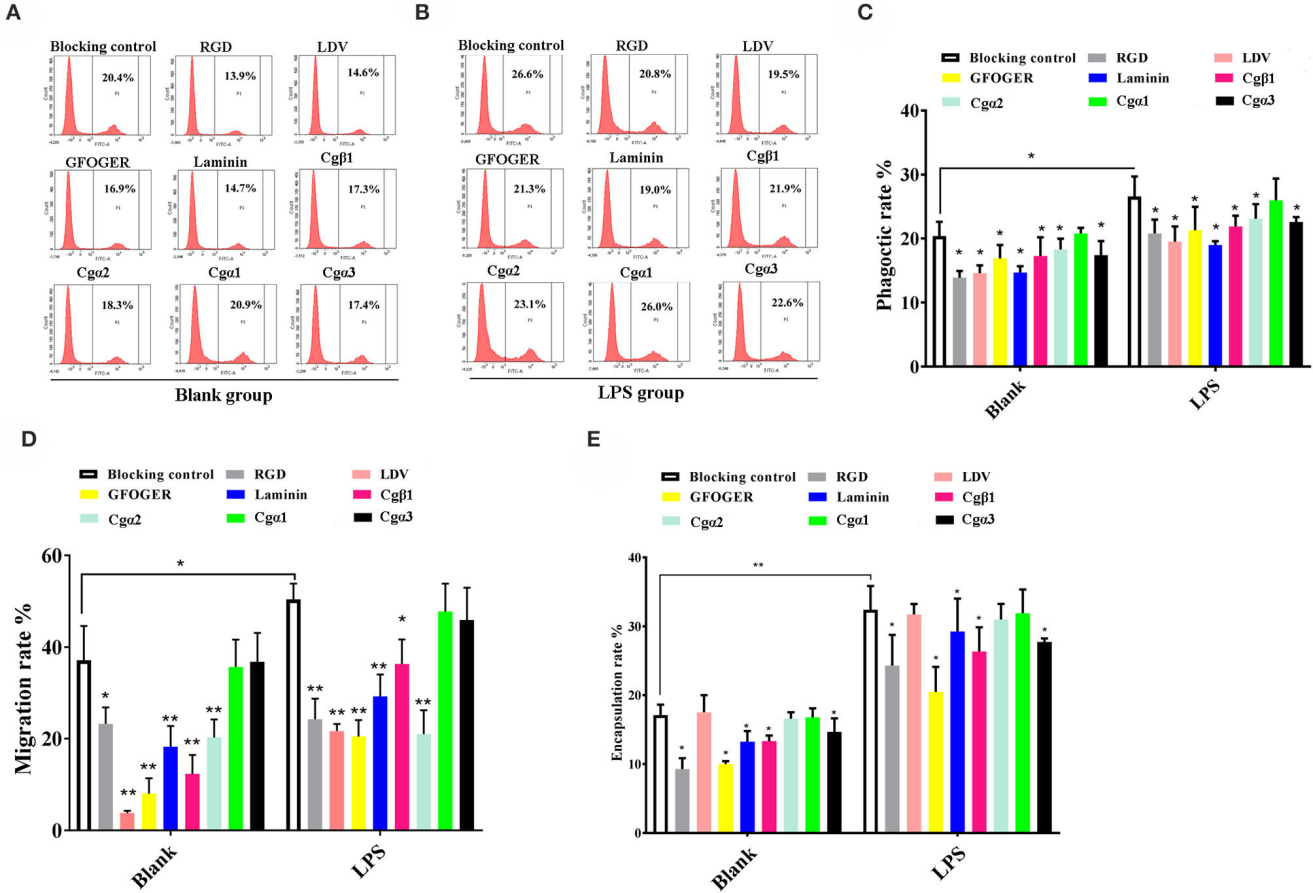
Oyster integrins are involved in hemocyte phagocytosis, migration, and encapsulation. Flow cytometry analysis shows the changes in phagocytosis rates of hemocytes in blank **(A)** and LPS-stimulated group **(B)** after the hemocytes are blocked by four integrin ligands including RGDCP, LDVCP, GFOGERCP, and laminin protein, and four different antibodies against oyster integrin proteins including *Cg*α1~3 and *Cg*β1. Column graphs about the changes of hemocyte phagocytosis rates **(C)**, encapsulation rates **(D)**, and migration rates **(E)** after the hemocytes are blocked by different integrin ligands and antibodies. Results are shown as mean ± SD (*n* = 3). *Stands for statistical significance (*p* < 0.05), and **stands for extremely statistical significance (*p* < 0.01), compared to the blocking controls.

In the blank control group that did not receive LPS stimulation, the phagocytosis of FITC-labeled *E. coli* by hemocytes were significantly blocked by the four integrin ligands (Figures 8A–C), and phagocytosis rates were decreased from 20.4% in blocking control to 13.9% in RGDCP group (*p* < 0.05), 14.6% in LDVCP group (*p* < 0.05), 16.9% in GFOGERCP group (*p* < 0.05), and 14.7% in laminin protein group (*p* < 0.05). The greatest inhibitory effect (31.9%) on phagocytosis was observed after the preincubation with RGDCP (Table S7). The results of additional blocking assays using the antibodies also showed that the antibodies to *Cgβ*1, *Cgα*2, and *Cgα*3 hindered hemocyte phagocytosis of FITC-labeled *E. coli*, and the associated phagocytosis rates were 17.2% (*p* < 0.05), 18.3% (*p* < 0.05), and 17.6% (*p* < 0.05), respectively, compared to that for the blocking control (20.4%). However, the inhibition was weaker than that observed in blocking assays using the four integrin ligands. Besides, the antibody used for blocking *Cgα*1 on hemocytes did not significantly inhibit the phagocytosis rate for FITC-labeled *E. coli*, compared to that of the blocking control (20.8 vs. 20.4%, *p* > 0.05). This suggested that *Cgα*1 may have been unnecessary for phagocytosis of *E. coli*. Furthermore, the antibodies to *Cgβ*1 inhibited hemocyte phagocytosis with the highest inhibition rate of 15.2%, compared to the other three α integrin antibodies (Table 3).

**Table 3:** The inhibition rates of specific integrin antibodies in the blocking assays.

	**α integrin representative**		**β integrin representative**	
	***Cg*α2**	***Cg*α1**	***Cg*α3**	***Cg*β1**
**Cell phagocytosis**	**✓**	**×**	**✓**	**✓**
Inhibition rate % (Blank)	10.3		14.7	15.2
Inhibition rate % (LPS)	13.2		15.0	17.7
**Cell migration**	**✓**	**×**	**×**	**✓**
Inhibition rate % (Blank)	45.3			66.8
Inhibition rate % (LPS)	58.3			27.9
**Cell encapsulation**	**×**	**×**	**✓**	**✓**
Inhibition rate % (Blank)			14.3	22.1
Inhibition rate % (LPS)			14.4	18.7

*Four different antibodies against oyster integrin proteins including Cgα1~3 and Cgβ1 have different inhibitory effects on hemocyte phagocytosis, migration, and encapsulation. “✓” or “ × ” indicates whether there is an inhibitory effect by integrin antibodies for cellular immune responses of the blank group (Blank, without LPS stimulation) or LPS-stimulated group (LPS)*.

In the LPS-challenged group, the phagocytosis rate observed for FITC-labeled *E. coli* increased to 26.6% (*p* < 0.05) after LPS stimulation compared to 20.4% in the blank control group (without LPS stimulation, Figures 8B,C). The highest inhibitory effect on the phagocytosis rates for hemocytes toward FITC-labeled *E. coli* was observed with laminin proteins in the blocking assays (28.6%, Table S7), which were performed using the four integrin ligands. The highest inhibition rate of 17.7% was observed with antibodies to *Cgβ*1, which inhibited hemocyte phagocytosis compared to the antibodies against the other three α integrin antibodies in blocking assays (Table 3). These results collectively indicated that certain RGDCP- and laminin-binding integrins were mainly involved in the phagocytosis of *E. coli*, and *Cgβ*1 was one of the key members to mediate hemocyte phagocytosis, but *Cgα*1 was not directly involved in phagocytosis.

#### Hemocyte Migration

Hemocyte migration in the blank oyster group (without LPS stimulation) was significantly hindered after the blocking by integrin ligands, including the RGDCP, LDVCP, GFOGERCP, and laminin proteins on hemocytes, with migration rates of 23.3% (*p* < 0.05), 3.8% (*p* < 0.01), 8.0% (*p* < 0.01), and 18.3% (*p* < 0.01), respectively, compared to that observed with the blocking control (37.1%, Figure 8D). LDVCP showed the highest inhibitory effect on migration, with an inhibition rate of 89.7% (Table S7). The antibodies to *Cgβ*1 and *Cgα*2 also reduced the hemocyte migration rates significantly to 12.3% (*p* < 0.01) and 20.3% (*p* < 0.01), respectively (Figure 8D), while the antibody to *Cgβ*1 displayed a higher inhibitory effect on migration, with inhibition rates of 66.8% (Table 3). The antibodies to *Cgα*1 (35.7 vs. 37.1%, *p* > 0.05) and *Cgα*3 (38.6 vs. 37.1%, *p* > 0.05) did not alter hemocyte migration rates, compared to that for the blocking control (Figure 8D). After LPS stimulation, hemocyte migration rate increased significantly from 37.1 to 50.6% (*p* < 0.05), compared to that for the blank group (Figure 8D). Both GFOGERCP and the antibody to *Cgα*2 showed the highest inhibitory effect on migration, with inhibition rates of 59.4 and 58.3% in blocking assays, respectively (Table 3 and Table S7). These results revealed that some of LDVCP- and GFOGERCP-binding integrins probably functioned as the crucial cell receptors to mediate hemocyte migration, and *Cgβ*1 and *Cgα*2 represented the important members, while *Cgα*1 and *Cgα*3 had no effect on hemocyte migration.

#### Hemocyte Encapsulation

In the blank control group that did not receive LPS stimulation, a significant inhibition of encapsulation rates for beads was observed after blocking by integrin ligands including the RGDCP, GFOGERCP, and laminin proteins, which were 9.3% (*p* < 0.05), 10.1% (*p* < 0.05), and 13.3% (*p* < 0.05), respectively, compared to the rate of 17.1% for the blocking control (Figure 8E). RGDCP inhibited hemocyte encapsulation with the highest inhibition rate of 45.8% (Table S7), while LDVCP did not inhibit hemocyte encapsulation, compared to that observed for the blocking control (17.5 vs. 17.1%, *p* > 0.05). The antibodies to *Cgβ*1 and *Cgα*3 also inhibited significantly the hemocyte encapsulation of beads at encapsulation rates of 13.4% (*p* < 0.05) and 14.7% (*p* < 0.05), respectively (Figure 8E). The antibody to *Cgβ*1 inhibited hemocyte encapsulation at a relatively higher inhibition rate of 22.1% (Table 3). However, the antibodies to *Cgα*2 (16.6 vs. 17.1%, *p* > 0.05) and *Cgα*1 (16.8 vs. 17.1%, *p* > 0.05) did not significantly inhibit hemocyte encapsulation (Figure 8E). After LPS stimulation, hemocyte encapsulation rate for beads increased from 17.1 to 32.4% (*p* < 0.01), compared to that for the blank group. GFOGERCP inhibited hemocyte encapsulation at a higher inhibition rate of 36.8% (Table S7) than that observed among the four integrin ligands, and antibody to *Cgβ*1 inhibited the hemocyte encapsulation rate by 18.7%, compared to that of the other three α integrin proteins in blocking assays using antibodies (Table 3). These results revealed that RGDCP- and GFOGERCP-binding integrins may also serve in hemocyte encapsulation of foreign particles, of which *Cgβ*1 was an important member, while *Cgα*1 and *Cgα*2 did not appear to be involved in hemocyte encapsulation.

## Discussion

The synergistic cooperation of multiple innate immune receptors completes the missing effects of adaptive immune responses by greatly increasing the diversity and specificity of immune responses, which might be considered as an essential characteristic of invertebrate innate immune responses (64). We show here a molecular characterization of all integrin family members in an invertebrate, the Pacific oyster, *C. gigas*, and show that the highly diversified oyster integrins, which have a broad ligand-binding spectrum, can mediate multiple cellular immune responses, including hemocyte phagocytosis, encapsulation, and migration via the function divisions and cooperation by various α and β integrins. The results provide solid evidence regarding the diversity and specificity of oyster immune responses.

### The Highly Diversified Oyster α/β Integrins Have Evolved Into Specific Phylogenetic Branches

Integrins exist widely in metazoans, ranging from sponges to humans (12, 14). Here we use a genome-wide screening for α*/*β integrins in the oyster. Although oyster integrins display more structural variability, such as FA-GAP region, sulfotransferase domain in α integrin proteins, and extra INB (or βI) domain in β integrin proteins, they all contain conserved domains, including the extracellular INA domain, Intα domain in the α integrin proteins, or extracellular INB domain in the β integrin proteins, and a short transmembrane domain and cytoplasmic domain in both α and β integrin proteins, as most integrins in other species (10, 65). Multiple sequence alignments of oyster integrins also reveals that seven of eight α integrin proteins and two of three β integrin proteins harbor the conserved motifs of GFFXR and NPX[Y/F] in their cytoplasmic region. As reported in human integrins, GFFXR and NPX[Y/F] are necessary and sufficient for integrin activation and integrin-mediated cellular process (66, 67). Based on the conserved structure of integrin, one α and nine β integrin candidates without transmembrane domain are excluded, and a total of eight α and three β oyster integrin genes are identified. The presence of more common integrin family members in *C. gigas*, compared to those in *D. melanogaster, C. elegans*, and *N. vectensis*, indicates that the integrin family has expanded in oysters. Many immune-related receptors from marine invertebrates, such as the toll-like receptor (TLR) family from the sea urchin (68), tumor necrosis factor (TNF) family from amphioxus (69), and TNF, lectin, and C1q protein families from oyster *C. gigas* (57, 70) are also observed to expand significantly. The large expansion of these immune-related gene families in oysters may be very helpful for their survival in pathogen-rich, ever-changing marine environments (70, 71). The expansion of integrins, which are important immune-related receptors, might be co-opted and integrated of available cellular mechanisms, to perform diverse functions and improve the survival of the oyster.

The hallmark structure of the INA domain in α integrin and the INB domain in β integrin are identified in oyster integrins, while a high degree of sequence variability is observed, upon comparing the inter- or intra-species integrin structures. It is reported that INA domains are composed of three immunoglobulin-like domains with more variations, and INB domains contain four to six β sheets, surrounded by six to eight α helices (9, 72). In the present study, the lengths of INA and INB domains from oyster integrins are highly varied and shorter than those in human integrins. Meanwhile, the similarity and identity of oyster integrin INA and INB domains are lower than those observed in human integrins. Therefore, it is not surprising that the spatial conformations of oyster integrins also show a high degree of variability. The structure of oyster α integrins can be classified into two types based on whether they contained α helices, whereas the typical and conserved thigh-to-calf regions are not observed in all oyster INA domains. The thigh-to-calf regions are present in all human α integrins and essential for integrin activation and signaling to support the upright conformation of the head region β-propellers (37, 73), which suggests that some important functions would be missing because of the lack of thigh-to-calf regions in oyster α integrins. The most visible structural variations observed in oyster INB domains is that they have some extra structures with different numbers of α helices and β sheets, in addition to the conserved central structure, which directly determines the binding ability of β integrins to extracellular ligands (74), and thus it increases the possibility for oyster β integrins to play distinct roles in ligand recognition and binding. Collectively, the domain composition and three-dimensional structures of oyster integrin family members are highly variable, which may provide the structural basis for their diverse and unique functions.

To identify the evolutionary features of oyster integrins, their phylogenetic relationships are analyzed by using integrins from the genomes of humans, insects, sea anemones, oysters, and nematodes. In the phylogenetic tree of α integrins, *Cg*α1 is clustered into laminin-binding receptors, and *Cg*α2 is clustered into RGD-binding receptors, and no oyster integrin family members present in αI-domain receptor, LDV-binding receptor, and PS3-type receptor branches. The αI-domain receptors and LDV-binding receptors can bind to the GFOGER motif and LDV motif, which are only identified in chordates, and invertebrates such as *D. melanogaster* and *C. elegans* have no such members (10, 75). This result is accordant with the domain composition analysis that α integrins from oyster and drosophila lack the αI-domains, which are deduced as invertebrate-lineage integrins. The αPS3 integrins have been observed to date only in insects, and their presence has been attributed to arthropod-specific radiations as deduced from their sequences (14). Six oyster α integrins are obviously clustered into one distinct group in a manner similar to that of αPS3 integrins in insects, and they exhibit significant differences in their structures and sequences, such as in *Cg*α5 and *Cg*α1 with extra FG-GAP regions, and *Cg*α5 with a special sulfotransferase domain. Consequently, these six oyster α integrins are identified as oyster-specific integrins. For the phylogenetic tree of β integrins, previous studies have classified β integrins into two major phylogenetic clades (vertebrate and invertebrate), including four distinct branches of chordate β, protostome β, insect βV, and early metazoan β, which underlines the phyla lineage-specific features of invertebrate β integrins (14, 76). Unexpectedly, three oyster β integrins are closely related to some β integrins from *D. melanogaster* and *A. gambiae* and clustered with the branch of insecta βV, while they are distant from the β integrin of mollusk, *B. glabrata*, suggesting the divergence between β integrins from *C. gigas* and *B. glabrata* has occurred during the evolution. *B. glabrata* is a freshwater species, while *C. gigas* is a marine mollusk (47, 77), so it make sense that the evolution of different invertebrate integrins with a distinct structural specificity may occur due to the various environments in which animals survive (7). Additionally, the similarity and identity of four oyster INB domains are 67.1 and 14.4%, which are in close proximity to the values of 69.6 and 14.9% obtained for eight INB domains in human β integrins. Oyster β integrins might have some conserved or important functions, because of which they are clustered into the insecta βV branch. Collectively, the oyster integrin family members have many specific domains, highly variable sequences, and diversified structures, which are possibly responsible for their uniqueness as species-specific members in a distinct evolutionary branch, as well as for providing the structural basis for them to perform diverse functions.

### Oyster Integrins Have a Broad Spectrum of ECM Ligands as Human Integrins

Integrins act as important cell receptors that transduce extracellular signals and mediate multiple cellular processes, which depends greatly on their ability to bind to a variety of ligands (31, 32). Their ligand-binding activities are determined by the extracellular domains from both α and β integrins, with α integrins playing a central role in ligand specificity (31, 67). The binding to ligands sometimes necessitates complex mechanisms that co-contribute to the binding of ligands and various integrins on the cell surface (78). This partly explained why SPR analysis fails to detect the binding of eight recombinant integrin proteins to any of the ECM ligands. While the SPR technique is a certified method to measure the special binding affinities between biological macromolecules, and the binding activities of *Cg*α1 with the laminin protein, *Cg*α2 with the laminin protein and RGDCP, and *Cg*α3 with GFOGERCP and RGDCP detected by SPR technique at least demonstrate the strong binding force between the three representative integrins and the typical ECM ligands.

Further experiments confirm that oyster integrins have a functional divisions and cooperation in ECM ligand binding. To be detailed, the RGD motif is mainly derived from ECM, including fibronectins, vitronectins, and certain laminins, which are well-known binding targets for integrins in various organisms (34). In humans, α5β1, α8β1, αVβ1, αVβ3, αVβ5, αVβ6, αVβ8, and αIIbβ3 belong to the RGD-binding receptors, and they can be involved in cellular immunity, cell motility, cell proliferation, and cell differentiation through the interaction with RGDCP (3). In invertebrates, the accumulating evidences have revealed that RGDCP can also bind hemocytes and inhibit the cell activities in *Botryllus schlosseri* (79), *Pacifastacus leniusculus* (26), *Lymnaea stagnalis* (39), *Mytilus trossulus* (41), etc., suggesting the existence of RGD-binding integrins in such animals. In the present study, *Cg*α2 is phylogenetically clustered with the RGD-binding receptor, and it expectedly shows the binding activity to the RGD peptide by SPR analysis. In addition, the laminin-binding receptor *Cg*α1, oyster-specific receptor *Cg*α3, and *Cg*β1 are also involved in the binding of hemocytes to RGDCP, according to the results of the blocking assays. It is assumed that most of oyster integrins can bind to RGDCP, and they may exhibit functional overlap and cooperation during the recognition of the RGD-containing proteins.

Laminins also represent an important class of integrin ligands in ECM that play crucial roles in integrin-mediated cell adhesion (80). Currently, the human integrins α3β1, α6β1, α7β1, and α6β4 are considered as the professional laminin receptors, which can specifically bind to laminins at different regions of laminin proteins (32, 36). Here, the oyster *Cg*α1 can directly bind to laminin proteins by SPR analysis, which is accordant with the phylogenetic analysis that *Cg*α1 is the only member present in laminin-binding receptor branch, so it is speculated that *Cg*α1 is a laminin receptor in oysters. In addition, previous studies have also shown that RGD-binding integrins can bind to laminins, because certain laminins possess the RGD motif (80), indicating the functional overlap of integrins during the binding of laminins. Similarly, the RGD-binding receptor *Cg*α2 can bind not only to RGDCP but also to laminin proteins, thereby demonstrating that *Cg*α2 also acts as a laminin receptor in oysters.

The GFOGER motif in certain collagens is recognized by collagen-binding integrins containing αI-domains, which are only present in vertebrates but not in invertebrates (38). It is worth noting that the I-domain is not unique to αI-domain integrins, because the homolog βI domain can be found in all β integrins (10). Hence, it has been argued that the human βI domain partly compensates for the ligand-binding activities of the αI domain (81). In the present study, the results of the hemocyte-ligand binding assay demonstrate that oyster hemocytes can bind to FITC-labeled GFOGERCP. SPR analysis also shows that the oyster-specific *Cg*α3 can bind to GFOGERCP. Further, the blocking assay shows that *Cg*β1 and *Cg*α3 can be involved in the binding of hemocytes to FITC-labeled GFOGERCPs. These results suggest that the lack of the functional αI-domain may be partly compensated by *Cg*β1 and *Cg*α3 in the oyster. Moreover, the results of α-β pairing analysis based on Pearson correlation coefficients also shows that *Cg*β1 and *Cg*α3 co-express in the same tissues during oyster stress or normal development and may constitute a functional integrin heterodimer, and that they possibly serve as a collagen receptor that collaboratively bind to GFOGER-containing proteins in oysters.

The LDV motif is minimally recognized in the alternatively spliced regions of fibronectin by human α4/α9-containing integrins, including α4β1, α4β7, and α9β1, which are generally evolutionarily lost in invertebrates (8, 14, 32). In the present study, oyster α integrins are also evolutionarily spaced in LDV-binding receptors. However, the oyster hemocytes can bind the FITC-labeled LDVCP in hemocyte-ligand binding assays. The results of blocking assays also display that the blocking of *Cg*β1 inhibits the binding of hemocytes to FITC-labeled LDVCP, indicating that *Cg*β1 can serve as an LDV-binding receptor and that the functional lack of LDV-binding receptors may be partly compensated by the presence of *Cg*β1 in oysters. As a result, *Cg*β1 is thought to compensate the functional lack of the αI-domain and LDV-binding integrins by binding to LDVCP and GFOGERCP, suggesting the indispensable roles of β integrins during the ligand binding process.

All above results together demonstrate that oyster integrins display a broad spectrum of binding-related activities with RGDCP, GFOGERCP, LDVCP, and laminins. Although oyster integrins evolutionarily lack the LDV-binding and αI-domain containing members, they can still bind to LDVCP and GFOGERCP, which possibly relies on the functional compensation of different members within the oyster integrin family. Therefore, it is strongly indicated that oyster integrin family members are highly functionally evolved and may have a distinct ligand-binding pattern from human integrins.

### Oyster Integrins Are Involved in Multiple Cellular Defense Processes With Sophisticated Cooperation and β-Dependence

Now only a few reports have shown that some single invertebrate integrins are involved in cell immune responses (28, 60, 82, 83), and the information of the most invertebrate integrins about their expression profiles, α-β integrin pairings, and distribution features on the hemocyte still remain largely unknown, which hinders the profound understanding of the underlying immune mechanism mediated by invertebrate integrins. In the present study, the expression profiles of oyster integrins from development stages, various tissues, and post-LPS stimulation are analyzed, and it is found that most oyster integrins are expressed in hemocytes, and up-regulated by LPS stimulation, which suggests their tendency to be involved in generating innate immune responses. Besides, α-β integrin pairings are predicted by the Pearson correlation analysis according to gene co-expression clusters (20). The results show that oyster integrins possibly form eight or more functional heterodimers. Among them, the three β integrin are arranged in the core position, and each of them can cooperate with two or three different α integrins, while there are only one α integrin (*Cg*α1) that can pair with both *Cg*β1 and *Cg*β2. Besides, the number of α-β integrin pairings in oysters is obviously more than in *N. vectensis* (15, 20), *D. melanogaster* (75), and *C. elegans* (75), indicating the varied α-β pairing patterns of oyster integrins that may enable them to mediate complex immune responses.

Circulating hemocytes are thought functionally analogous to vertebrate leukocytes and play crucial roles during host defense in oyster (84, 85); therefore, the cellular localization of different integrins is observed. For instance, *Cg*α1 appears to occur on both the smaller and larger cytoplasmic-nucleo ratio hemocytes with uniform expression levels, which may be related to its co-expression with both *Cg*β1 and *Cg*β2 that may make it expressed on at least two different hemocyte subpopulations. Similarly, the localization of *Cg*β1 on different hemocyte subpopulations is probably related to its broad ECM ligand-binding ability and cooperation with three α integrins, especially that the co-localization of *Cg*β1 and *Cg*α3 on the hemocyte subpopulation with a smaller cytoplasmic-nucleo ratio is accordant with the results that these two integrins may function as a pair in some hemocytes. Hemocytes with a different cytoplasmic-nucleo ratio, cell size, or cytoplasmic granules play different immune functions in oysters (48, 84). For example, granulocytes with a relatively bigger cytoplasmic-nucleo ratios and cell sizes represent the main immunocompetent hemocytes with higher antimicrobial activities, while the semigranulocytes and agranulocytes does not represent the main immune killing hemocytes in *C. gigas* (85). Therefore, the distribution of integrin family members on specific hemocyte subpopulations suggests their different manners in the mediation of immune responses in oysters.

Our experiments further confirm that oyster integrins indeed participate in multiple cellular immune responses of hemocyte phagocytosis, migration, and encapsulation with a clear function division and sophisticated cooperation. In ligand-blocking experiments, RGDCP and laminin protein show relatively higher levels of inhibitory effects on hemocyte phagocytosis for *E. coli*. LDVCP and GFOGERCP exert relatively higher level of inhibitory effects on hemocyte migration, and RGDCP and GFOGERCP show relatively higher levels of inhibitory effects on hemocyte encapsulation for large foreign agents. The results from the inhibitory effects of different integrin antibodies on various immune responses also reveal that *Cg*β1, *Cg*α2, and *Cg*α3 may be responsible for hemocyte phagocytosis, but the presence of *Cg*α1 seems to be unnecessary for this process. *Cg*β1 and *Cg*α2 induce hemocyte migration, but *Cg*α1 and *Cg*α3 seem to be inactive. *Cg*β1 and *Cg*α3 participate in hemocyte encapsulation, while *Cg*α1 and *Cg*α2 are not required. Meantime, it is also found that various integrin members exhibit synergistic cooperation, in order to mediate complex cellular immune responses as in vertebrates (3, 17, 67). For instance, the human integrins αLβ2, αDβ2, αXβ2, and αMβ2 cooperate to enable the migration of human leukocytes to infection sites, for immune surveillance and host defense (10). In the present study, *Cg*α2, *Cg*α3, and *Cg*β1 are involved in hemocyte phagocytosis, *Cg*α2 and *Cg*β1 are involved in hemocyte migration, and *Cg*α3 and *Cg*β1 participate in hemocyte encapsulation. These findings suggest that the entire oyster integrin family members probably have tightly integrated with the innate immune system, by selecting the specific members to mediate different immune responses in a sophisticated cooperation manner.

In mammals, integrins exert essential functions in cellular immunity, which greatly relies on several αI-domain-containing integrins, mainly leukocyte-specific receptors (17). In invertebrates, all integrins lack the αI-domain (10). It is uncertain now whether the βI-domain compensates the immune functions, though more and more reports have revealed the indispensable role of β integrins in invertebrate immune responses. For instance, the β1-like integrin identified on the surface of *L. stagnalis* hemocytes can modulate the phosphorylation of focal adhesion kinase, phagocytosis, and cell spreading (39). The *D. melanogaster* βV (27), *A. gambiae* BINT2 (83), and a *C. gigas* β integrin (25) have been found to mediate the phagocytosis of bacteria. A shrimp β integrin is confirmed to directly recognize the WSSV envelope protein VP187 with the RGD motif (86). In our study, structure analysis reveals that oyster β integrins possess some variable structures, which may support them to recognize and bind multiple ligands. Besides, α-β pairing prediction also reveals that β integrins are arranged in the core position, which makes them more flexible to cooperate with different α integrins to activate various cellular immune responses. Function analysis confirm that oyster *Cg*β1 is the only member of the tested integrins appears to be involved in the all detected cellular immune responses, and the antibody to *Cg*β1 exhibits a relatively higher level of inhibitory effects on these immune responses than the other three α integrin antibodies. Hence, it is reasonable to infer that the involvement of oyster integrin in multiple cellular immune responses is largely dependent on the presence of β integrins.

## Conclusions

**Figure 9:**
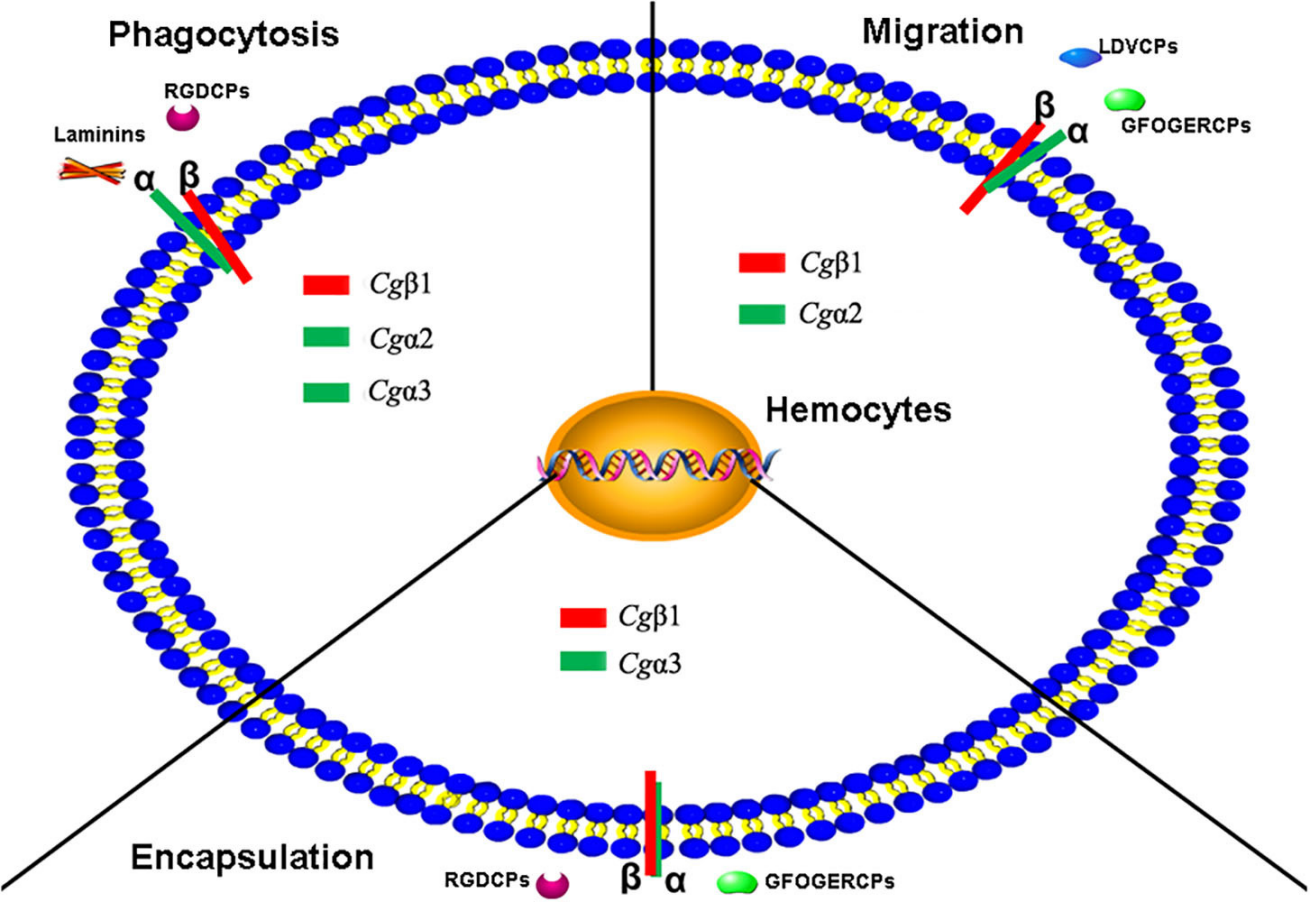
The schema diagram for the various immune responses mediated by oyster integrins with the possible α-β pairings and the binding to multiple ECM ligands. Oyster integrin representatives of *Cg*α1~3 and *Cg*β1 bind to RGDCP, LDVCP, GFOGERCP, and laminin protein with clear functional divisions and are deduced to be involved in cellular immune responses of hemocyte phagocytosis, migration, and encapsulation.

In summary, eight α and three β integrins are identified from the oyster *C. gigas* genome. Oyster integrins contain conserved domains but also exhibit a high degree of sequence and structural variability compared to those in *H. sapiens* and *D. melanogaster*. Eight α integrins are evolutionarily present in RGD-binding receptors, laminin-receptors, and mainly grouped as oyster-specific receptors. Three β integrins are phylogenetically similar to insect βV branch and are distantly related to one β integrin from the mollusk *B. glabrata*, indicating that oyster α and β integrins may have different evolutionary histories, and both have developed into distinct evolutionary branches. The study on the functional characteristics of oyster integrins involved in ligand-binding activities and cellular immune responses demonstrates that oyster integrins can bind to multiple ECM ligands, including RGDCP, LDVCP, GFOGERCP, and the laminin protein and are involved in hemocyte phagocytosis, migration, and encapsulation, in a sophisticated cooperation pattern and β-dependence (Figure 9). The present results may enrich the evolutionary theory of the integrin family and present solid evidence regarding the functional characteristics of the integrin family. It also provides insights into the characteristics of the innate immune system, which is dominated by the delicately synergistic cooperation of multiple immune receptors in invertebrates.

## Data Availability Statement

Publicly available datasets were analyzed in this study. This data can be found here: oyster_v9, http://ensemblgenomes.org. Other raw data supporting the conclusions of this article will be made available by the authors, without undue reservation, to any qualified researcher.

## Ethics Statement

All experiments involving animals reported in this study were approved by the Ethics Committee of the Institute of Oceanology, Chinese Academy of Sciences.

## Author Contributions

ZLv, LQ, LS, and LW conceived and designed the experiments and wrote the manuscript. ZLv performed the experiments and analyzed the data. LQ and QL contributed reagents, materials, and analysis tools. ZLv, WW, and ZLi contributed to the discussion. All authors read and approved the final manuscript.

## Conflict of Interest

The authors declare that the research was conducted in the absence of any commercial or financial relationships that could be construed as a potential conflict of interest.

## Abbreviations

αPS1-5: position-specific type 1–5 alpha integrin
αI domain: “inserted” domain of alpha integrin
βI domain: “inserted” domain of beta integrin
BINT2: beta2 integrin
BSA: bovine serum albumin
βV: betaV integrin
DAPI: 4′,6-diamidino-2-phenylindole
ECM: extracellular matrix
EGF: epidermal growth factor
FBS: fetal bovine serum
FG-GAP repeats: phenylalanine-glycine-glycine-alanine-proline repeats
FITC: fluorescein isothiocyanate
FPKM: fragments per kilobase million
FSC-A: forward scatter area
GFOGER: glycine-phenylalanine-hydroxyproline-glycine-glutamate-arginine
GFOGERCP: GFOGER-containing peptide
HEPES: hydroxyethyl piperazine ethylsulfonate
HMM: hidden Markov model
Intα: integrin alpha repeats
INA domain: integrin_alpha2 domain
INB domain: integrin beta domain
LDV: leucine-aspartic acid-valine
LDVCP: LDV-containing peptide
LPS: lipopolysaccharide
RGD: arginine-glycine-aspartate
RGDCP: RGD-containing peptide
RPKM: reads per kilobase million
RU: response unit
SSC-A: side scatter area
TLR: toll-like receptor
TNF: tumor necrosis factor
VCBS domain: repeat domain in Vibrio, Colwellia, Bradyrhizobium, and Shewanella
vWA: von Willebrand factor type A
WSSV: white spot syndrome virus.
