# Supplementary material for: The Members of the Highly Diverse Crassostrea gigas Integrin Family Cooperate for the Generation of Various Immune Responses
Source: Front Immunol. 2020 Jul 23;11:1420. doi: 10.3389/fimmu.2020.01420 (PMC7390872; doi:10.3389/fimmu.2020.01420)
Supplement: Supplementary file 6 [file Table_6.docx]

**Table S6.** **Analysis of oyster integrin α-β pairing based on trasncriptome data with Pearson correlation coefficients.**

|  | **Developmental stage** | | **Tissue distribution** | | **LPS stimulation** | |
| --- | --- | --- | --- | --- | --- | --- |
| **β** | **α** | **Pearson' R** | **α** | **Pearson' R** | **α** | **Pearson' R** |
|  | CG1_10010727 | 0.8436 |  |  | CG1_10008246 | 0.9831 |
| CG1_10012180 | CG1_10008246 | 0.9335 |  |  |  |  |
|  | CG1_10023513 | 0.8235 | CG1_10023513 | 0.8879 |  |  |
|  | CG1_10013155 | 0.3535 |  |  |  |  |
| CG1_10012179 | CG1_10005638 | 0.8314 | CG1_10005638 | 0.6913 | CG1_10005638 | 0.9212 |
|  |  |  |  |  | CG1_10021391 | 0.4845 |
|  | CG1_10005638 | 0.1851 | CG1_10005638 | 0.0119 |  |  |
| CG1_10014761 | CG1_10013155 | 0.1449 |  |  | CG1_10013155 | 0.9958 |

Footnote: This table showed the whole positive Pearson correlation coefficients of co-expression of oyster α and β integrin genes based on RNA-seq data derived from developmental stages, tissue distribution, and LPS stimulation, respectively.
