# Supplementary material for: The Members of the Highly Diverse Crassostrea gigas Integrin Family Cooperate for the Generation of Various Immune Responses
Source: Front Immunol. 2020 Jul 23;11:1420. doi: 10.3389/fimmu.2020.01420 (PMC7390872; doi:10.3389/fimmu.2020.01420)
Supplement: Supplementary file 7 [file Table_7.docx]

**Table S7. The inhibition rates of integrin ligands in blocking assays.**

| **Integrin ligands** | **RGDCP** | **LDVCP** | **GFOGERCP** | **Laminin proteins** |
| --- | --- | --- | --- | --- |
| **Cell phagocytosis** | **√** | **√** | **√** | **√** |
| Inhibition rate % (Blank) | 31.9 | 28.4 | 17.2 | 27.9 |
| Inhibition rate % (LPS) | 21.8 | 26.7 | 19.9 | 28.6 |
| **Cell migration** | **√** | **√** | **√** | **√** |
| Inhibition rate % (Blank) | 37.3 | 89.7 | 78.4 | 66.8 |
| Inhibition rate %  (LPS) | 51.8 | 56.9 | 59.4 | 27.9 |
| **Cell encapsulation** | **√** | **×** | **√** | **√** |
| Inhibition rate % (Blank) | 45.8 |  | 41.4 | 22.6 |
| Inhibition rate % (LPS) | 25.1 |  | 36.8 | 9.7 |

Footnote: “√”or “×” was used to indicate whether there was inhibitory effect for integrin ligands in cellular immune responses of of blank (without LPS stimulation) or LPS group oysters.
