# Supplementary material for: The Members of the Highly Diverse Crassostrea gigas Integrin Family Cooperate for the Generation of Various Immune Responses
Source: Front Immunol. 2020 Jul 23;11:1420. doi: 10.3389/fimmu.2020.01420 (PMC7390872; doi:10.3389/fimmu.2020.01420)
Supplement: Supplementary file 8 [file Image_1.pdf]

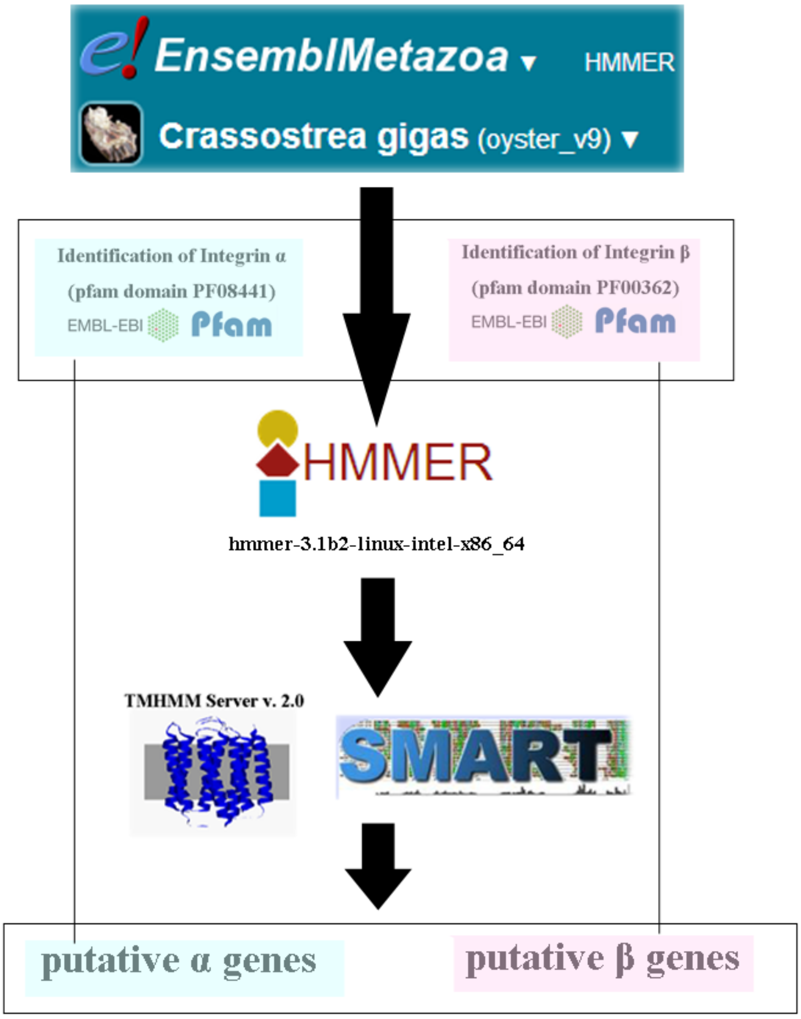

Figure S1. The pipeline for screening integrin genes from *C. gigas*. The α and β integrin gene candidates were retrieved from the *C. gigas* genome (oyster_v9, http://ensemblgenomes.org) using HMMER3.1 software with a multi-sequence alignment algorithm, and with default parameters using the INA domain (PF08441) or INB domain (PF00362) as templates. The domain prediction analysis was performed to verify the putative integrin family members using SMART program (http://smart.embl-heidelberg.de/).

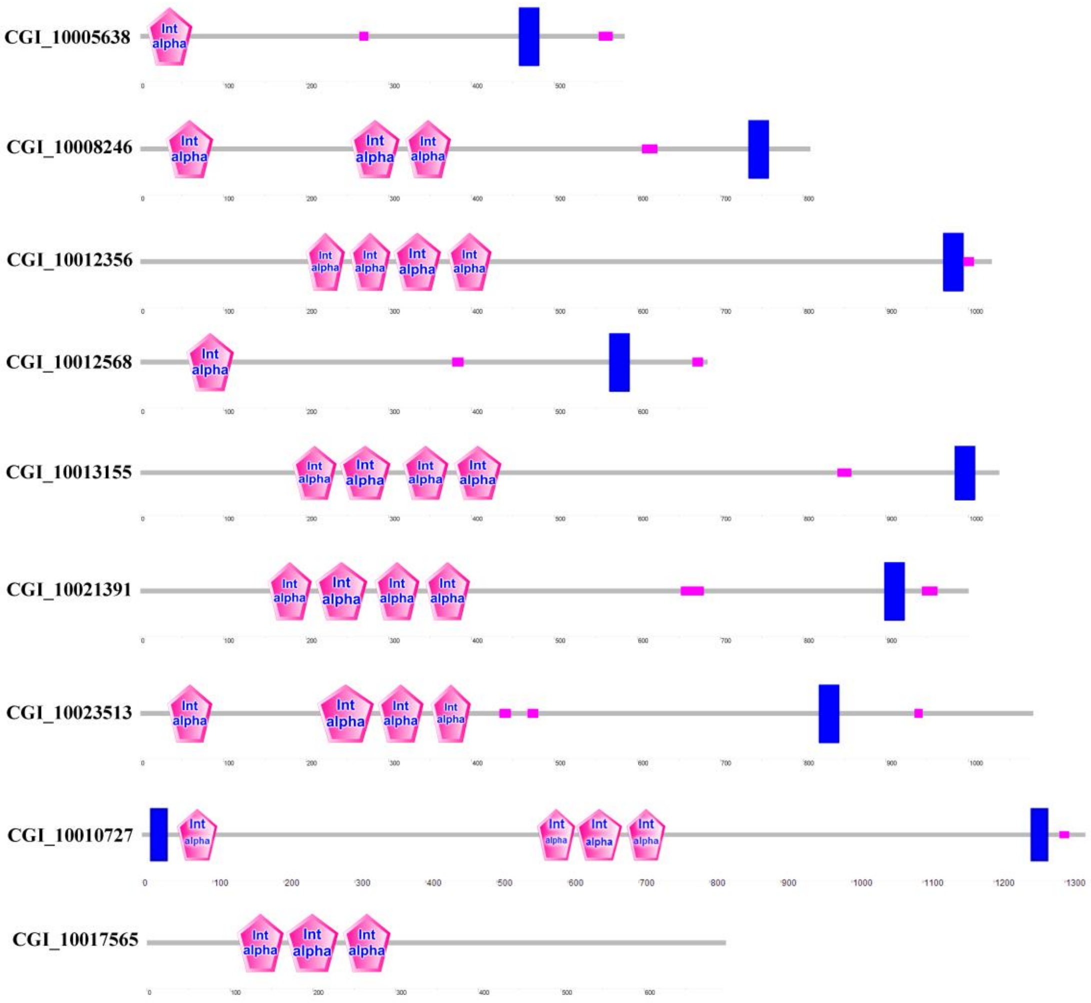

Figure S2. SMART analysis of protein domain of oyster nine α integrin candidates.

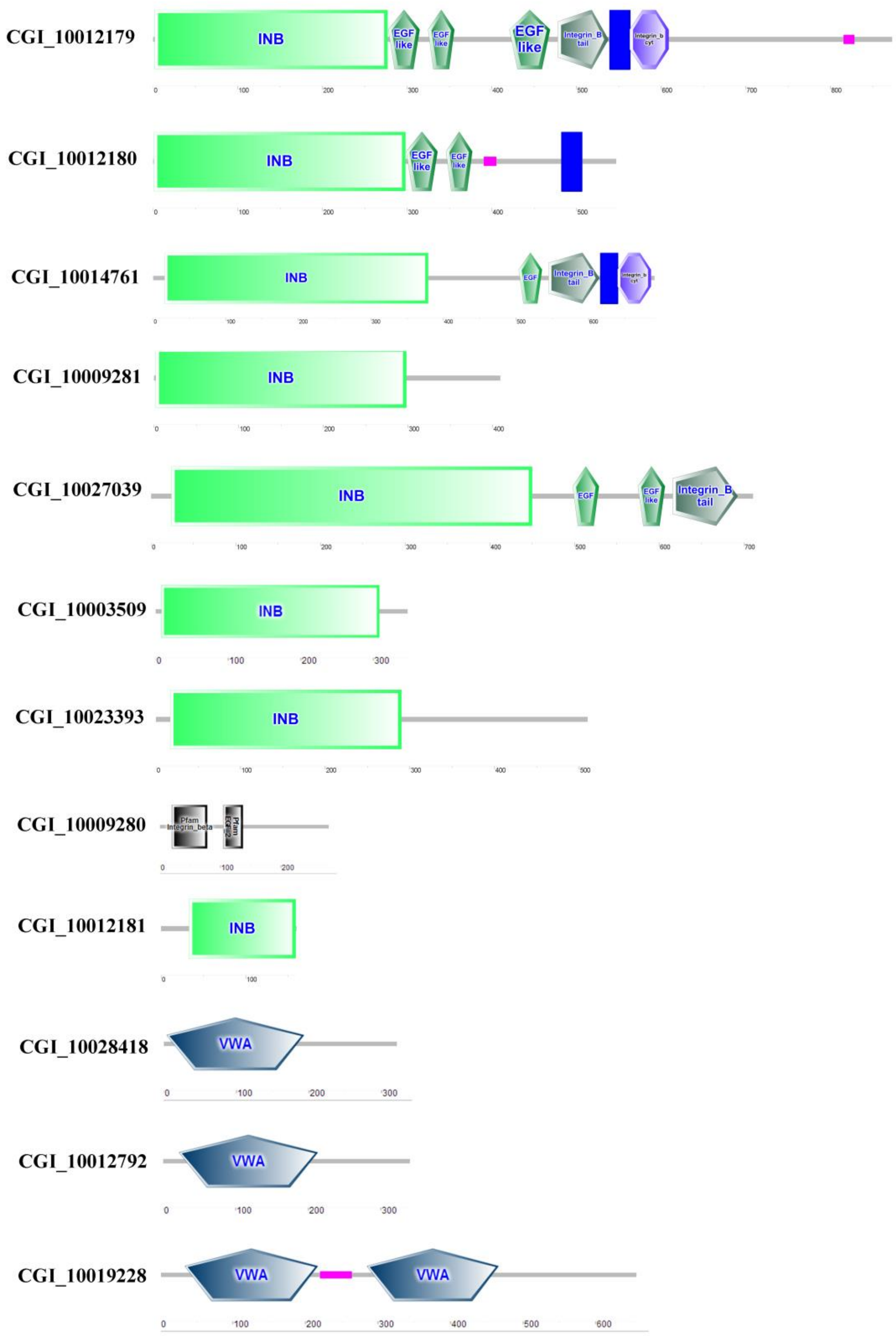

Figure S3. SMART analysis of protein domain of oyster twelve β integrin candidates.

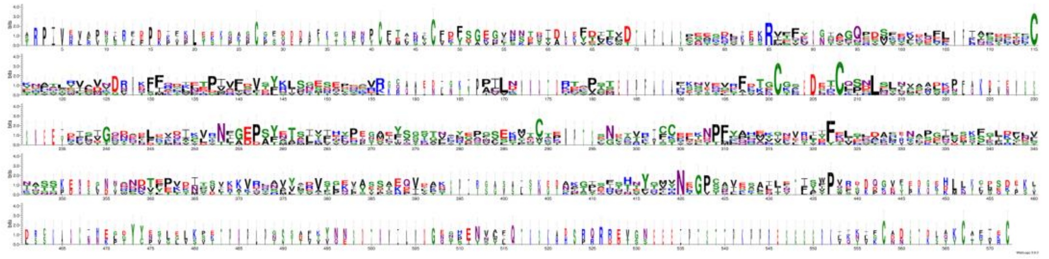

Figure S4. WebLogo analysis of INA domains from eight oyster α integrins.

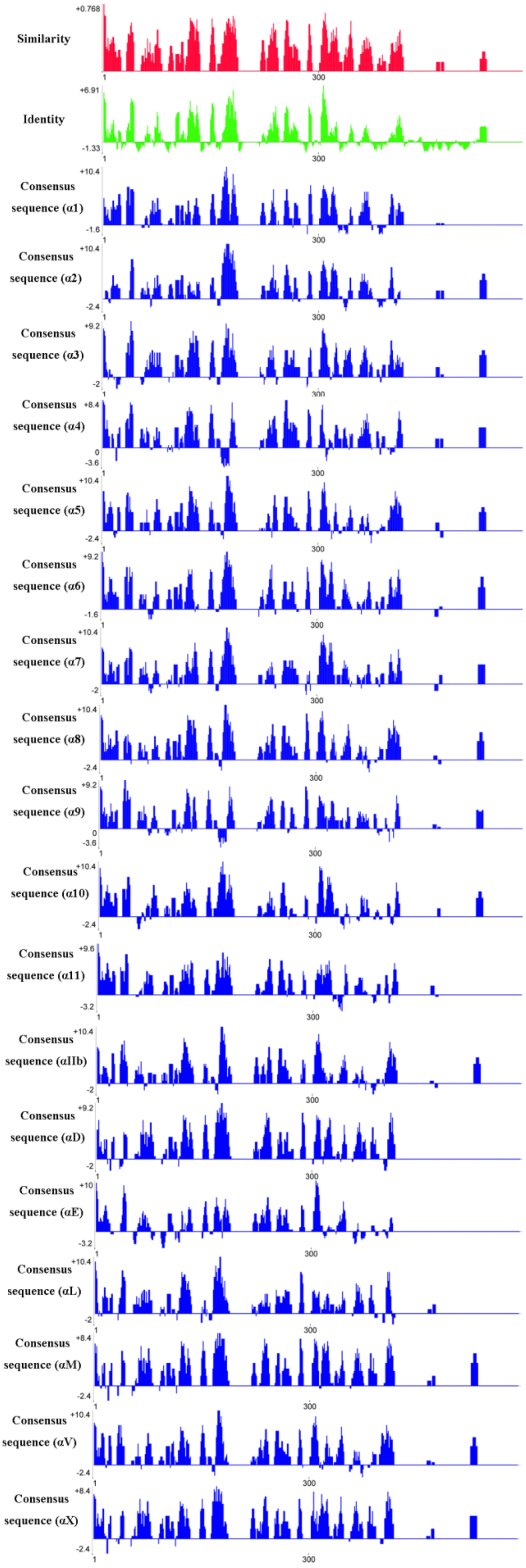

Figure S5. The identity and similarity of INA domains in human α integrins. The sequence alignment analysis is conducted by VectorNTI 10 software to reveal the conserved and similar amino acid sites among human integrin INA domains. The red and green histograms show the overall similar and conserved amino acid sites of INA domains in human α integrins, and the blue histograms show the conserved amino acid sites of each INA domain of human α integrins.

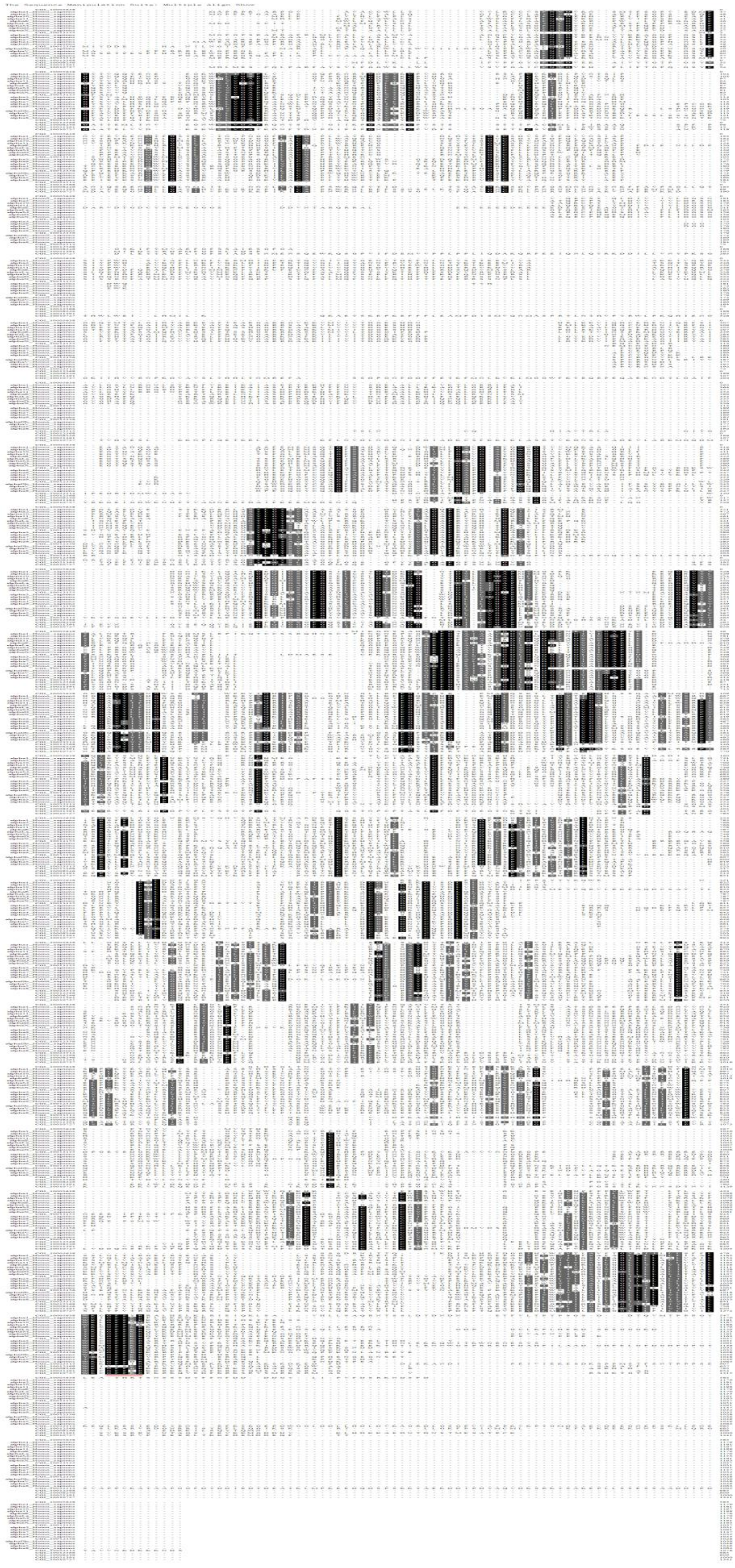

Figure S6. Multiple sequence alignments of full-length amino acids anlysis for oyster and human α integrins. The conserved GFFXR motif is marked by the red double underline.

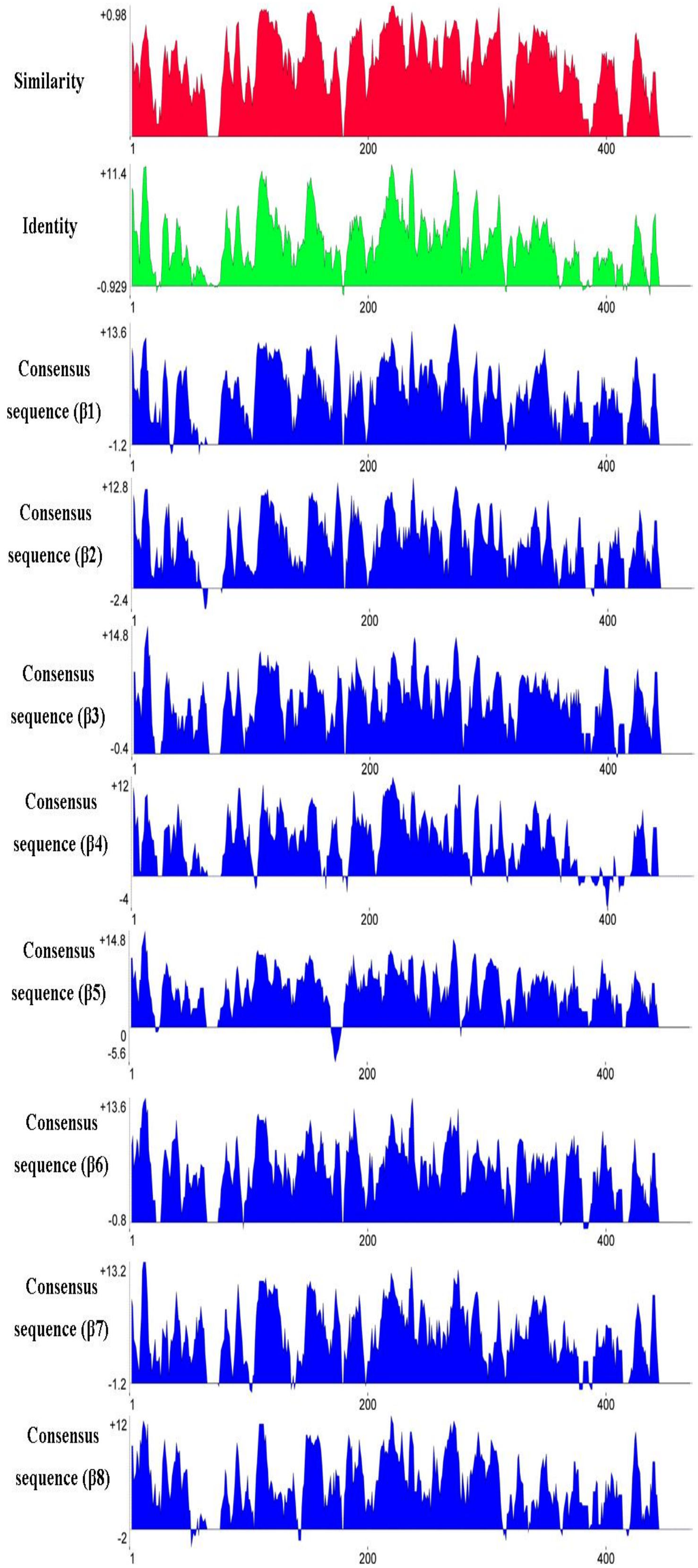

Figure S7. The identity and similarity of INB domains in human β integrins. The sequence alignment analysis is conducted by VectorNTI 10 software to reveal the conserved and similar amino acid sites among human integrin INB domains.The red and green histograms show the overall similar and conserved amino acid sites of INB domains in human β integrins, and the blue histograms show the conserved amino acid sites of each INB domain of human β integrins.

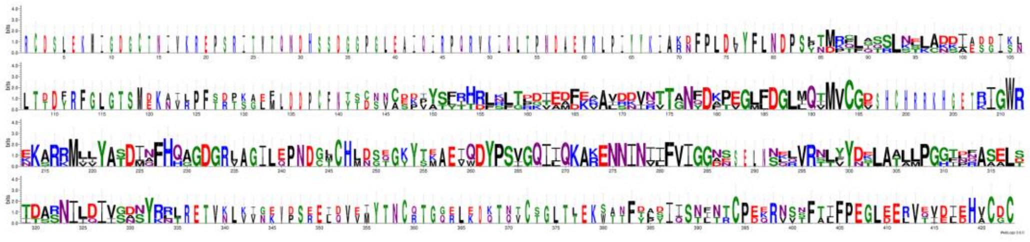

Figure S8. WebLogo analysis of INB domains from eight oyster β integrins.

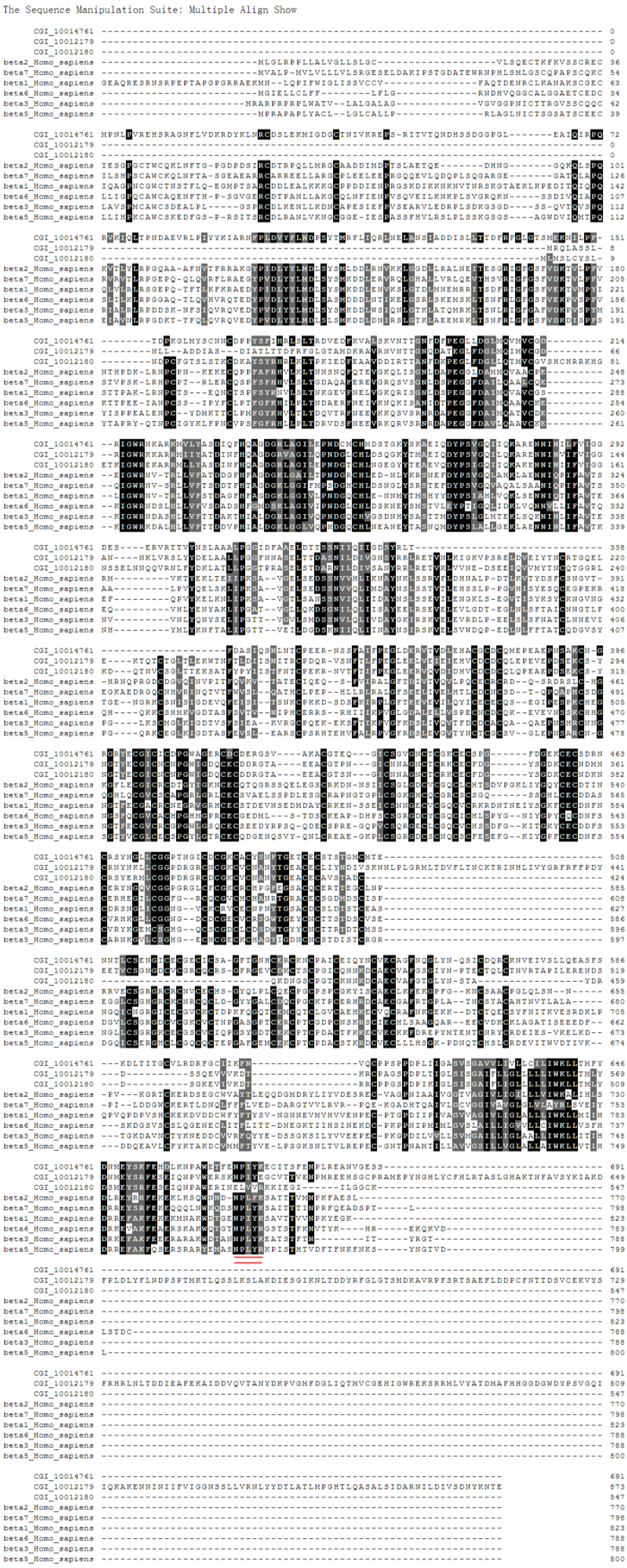

Figure S9. Multiple sequence alignments of full-length amino acids analysis for oyster and human β integrins. The conserved NPXY/F motif is marked by the red double underline.

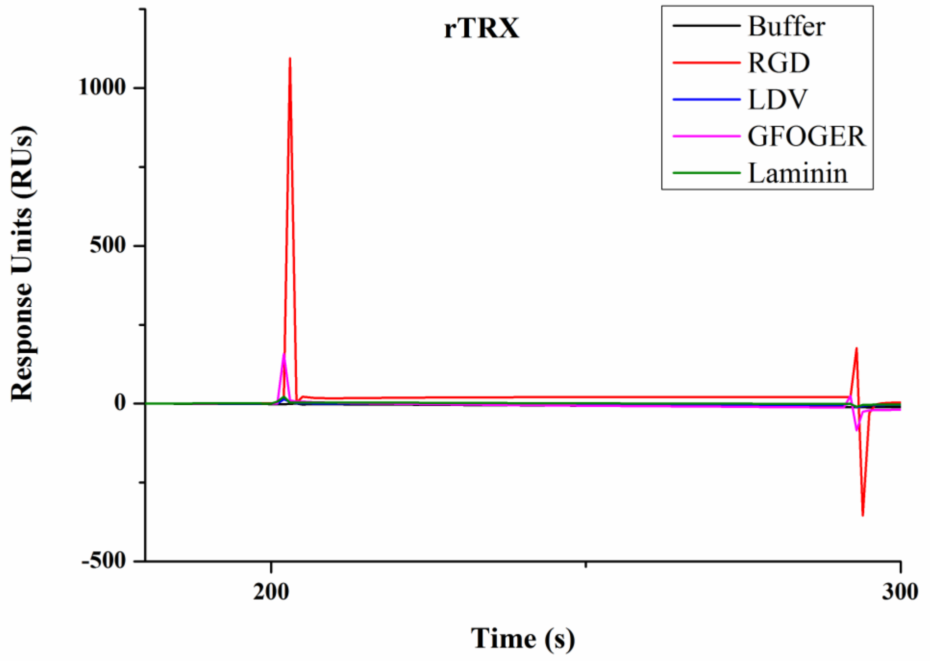

Figure S10. The binding of rTRX to integrin ligands. Recombinant TRX protein is used as the negative control, and the binding reactions of rTRX to RGDCP, LDVCP, GFOGERCP, and laminin protein are <10 RUs.
